# Machine Learning Density Functionals from the Random-Phase Approximation

**DOI:** 10.1021/acs.jctc.3c00848

**Published:** 2023-10-06

**Authors:** Stefan Riemelmoser, Carla Verdi, Merzuk Kaltak, Georg Kresse

**Affiliations:** † Faculty of Physics and Center for Computational Materials Science, University of Vienna, Kolingasse 14-16, A-1090 Vienna, Austria; ‡ Vienna Doctoral School in Physics, University of Vienna, Boltzmanngasse 5, A-1090 Vienna, Austria; ¶ School of Physics, The University of Sydney, Sydney, New South Wales 2006, Australia; § School of Mathematics and Physics, The University of Queensland, Brisbane, Queensland 4072, Australia; ∥ VASP Software GmbH, Sensengasse 8/12, A-1090 Vienna, Austria

## Abstract

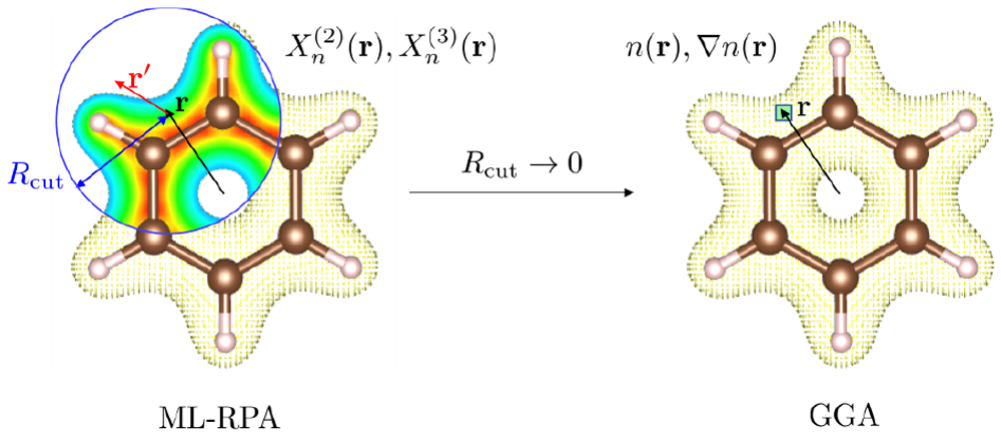

Kohn–Sham density functional theory (DFT) is the standard method for first-principles calculations in computational chemistry and materials science. More accurate theories such as the random-phase approximation (RPA) are limited in application due to their large computational cost. Here, we use machine learning to map the RPA to a pure Kohn–Sham density functional. The machine learned RPA model (ML-RPA) is a nonlocal extension of the standard gradient approximation. The density descriptors used as ingredients for the enhancement factor are nonlocal counterparts of the local density and its gradient. Rather than fitting only RPA exchange-correlation energies, we also include derivative information in the form of RPA optimized effective potentials. We train a single ML-RPA functional for diamond, its surfaces, and liquid water. The accuracy of ML-RPA for the formation energies of 28 diamond surfaces reaches that of state-of-the-art van der Waals functionals. For liquid water, however, ML-RPA cannot yet improve upon the standard gradient approximation. Overall, our work demonstrates how machine learning can extend the applicability of the RPA to larger system sizes, time scales, and chemical spaces.

## 1 Introduction

For over half a century, generations of researchers have been looking to find ever better approximations for the elusive exchange-correlation (xc) functional of Kohn–Sham density functional theory (DFT).^1,2^ The Hohenberg–Kohn theorems guarantee this exchange-correlation functional to be a universal functional of the electronic density *n*; in other words, there exists a map *n* → *E*_xc_[*n*].^3^ However, the exact functional is of complex nonlocal form and in general unknown. Common approximations for the exchange-correlation energy are built on the principle of near-sightedness:^4^ in the local density approximation (LDA),^5^ the exchange-correlation energy density is approximated to be locally that of a homogeneous electron gas with the same density.

To improve the accuracy of the approximation, one can add more nonlocal information by climbing up Jacob’s ladder of DFT.^6^ Next to the local density, one includes its gradient |∇*n*| as an ingredient to the exchange-correlation functional (generalized gradient approximation or GGA). Going beyond the GGA, more nonlocal information is added in the form of the kinetic energy density (meta-GGA), and fractions of exact exchange (hybrids). However, strictly speaking, most meta-GGA and hybrid functional approaches deviate from “pure” Kohn–Sham DFT, as the ingredients go beyond the electronic density alone.^7^ A different route beyond GGA are nonlocal van der Waals (vdW) functionals,^8,9^ which account for pairwise dispersion interactions between densities *n*(**r**) and *n*(**r**′). As the ingredients are only the electronic density and its gradient at points **r** and **r**′, the nonlocal vdW method stays within pure KS-DFT.

Given ingredients **X** for the exchange-correlation functional, one still has to find the actual functional form, that is the map **X** → *E*_xc_[**X**]. In the analytical approach developed by Perdew and others,^10−12^ those maps are found by satisfying exact constraints. The empirical approach pursued by Becke and others^13−16^ optimizes a small number of adjustable parameters to experimental data or higher level theory. Broadly speaking, the analytical functionals are more universally applicable, whereas the empirical approach can achieve higher accuracy for systems similar to the ones represented in their respective training sets.^2^ This comes at the cost of worse performance for systems not represented by these training sets. For example, the widely used B3LYP functional,^17^ whose parameters have been optimized for small main group molecules, performs well for main group chemistry but struggles when applied to extended systems^18^ and transition metal chemistry.^19^

Recently, machine learning (ML) techniques have been taking the empirical approach to its extreme.^20−23^ Not limited by human parametrizations, machine learning approaches can optimize the maps **X** → *E*_xc_[**X**] using complicated nonlinear functional forms. The first attempt of creating an ML density functional goes back to Tozer et al.,^24^ an effort which culminated in the development of the HTCH functional (Hamprecht–Tozer–Cohen–Handy, a popular GGA functional).^25^ The full potential of ML approaches has been demonstrated in pioneering work by Burke and co-workers, who showed that orbital-free density functionals can be learned from the full nonlocal density.^26,27^ Their approach was later applied to standard Kohn–Sham DFT, enabling molecular dynamics simulations of single molecules with chemical accuracy.^21,28,29^ Though this is very impressive, these ML-DFT functionals are tailor-made for this specific purpose and have to be retrained for every new molecule. A different approach was taken by Nagai et al.,^20^ who complemented meta-GGA ingredients by a nonlocal density descriptor, achieving remarkable accuracy for a large molecular test set with training data from only three molecules. For a broader discussion of different ML-DFT approaches, we refer also to the review of Schmidt et al.^30^

In the current work, we propose an approach to construct machine learned density functionals from the random-phase approximation (RPA, a high-level functional from the top of Jacob’s ladder^6^). We adapt the power spectrum representation of atomic environments used for machine learned force fields^31,32^ (MLFFs) to construct ingredients for ML-DFT. We show that these ingredients can be considered to be a nonlocal extension of GGA. In MLFFs, data efficiency can be improved by training not only on energies alone but rather also on atomic forces.^33^ Analogously, derivative information in DFT can be supplied via the exchange-correlation potentials,
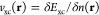
1However, obtaining accurate exchange-correlation potentials from beyond GGA functionals is generally difficult, and aside from the early work of Tozer et al., this approach has only been applied to simple model systems.^34−36^ Here, we supply such derivative information by using our recent implementation of the optimized effective potential method to obtain exchange-correlation potentials from the RPA.^37^ We demonstrate our method by fitting ML-RPA to diamond and liquid water and show that it enables larger scale RPA calculations. ML-RPA achieves its speed-up via bypassing the optimized effective potential equation, substituting the complicated RPA exchange-correlation functional with pure KS-DFT. Further, our efficient plane-wave implementation brings the system size scaling of ML-RPA down to that of standard DFT. Finally, our approach enables self-consistent calculations, force and stress predictions, and even molecular dynamics simulations for molecules, solids, and their surfaces. The rest of this article is organized as follows. Sec. 2 introduces the ML-RPA formalism. In Sec. 3, we briefly discuss the optimized effective potential method and comment on the issue of electronic self-consistency. Results are presented in Sec. 4 and discussed in Sec. 5. Conclusions are drawn in Sec. 6.

## 2 ML-RPA Formalism

### 2.1 Representation of the Electronic Density

We adapt the power spectrum representation of atomic environments^31^ to electronic densities as follows. The electronic density around each real-space grid point **r** is expanded into radial basis functions ϕ_*nl*_ (described in Supplementary Sec. S1) times real spherical harmonics *Y*_*l*_^*m*^
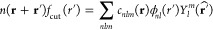
2The cutoff function *f*_cut_ puts emphasis on nearby densities (*r*^′^ ≤ *R*_cut_), following Kohn’s principle of nearsightedness.^4,38^ Here, we use a cutoff radius of *R*_cut_ = 1.5 Å. The expansion coefficients *c*_*n*00_ are the equivalents of rotationally invariant two-body descriptors used in MLFFs, thus we write *X*_*n*_^(2)^ = *c*_*n*00_. In the limit of small cutoffs *R*_cut_, the *X*_*n*_^(2)^ reduce to the local density
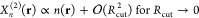
3as shown in Supplementary Sec. S1. It is interesting that the well-known weighted density approximation^39^ can be viewed as the special case where only a single two-body descriptor is taken, compare also the nonlocal density descriptor introduced by Nagai et al.^20^

Further, angular information is accounted for by forming rotationally invariant combinations of the *l* = 1 expansion coefficients to construct additional density descriptors *X*_*n*_^(3)^, similar to the three-body descriptors in MLFFs,
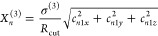
4Here, σ^(3)^ is an ML hyperparameter that weighs the *X*_*n*_^(3)^ relative to the *X*_*n*_^(2)^.^40^ For small cutoffs, the *X*_*n*_^(3)^ reduce to the local gradient
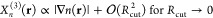
5as demonstrated in Supplementary Sec. S1. In summary, an exchange-correlation functional with *X*_*n*_^(2)^ and *X*_*n*_^(3)^ as its ingredients can be considered as a *nonlocal extension of the generalized gradient approximation*. Here, we use 4 radial basis functions; thus, in total we have 4 + 4 = 8 density descriptors. A representation based on nonlocal convolutions of the electronic density has been previously suggested by Lei and Medford,^41^ who also showed how the representation can be systematically completed by adding higher body-order descriptors. Likewise, representations of atomic environments for MLFFs can be completed via moment tensor potentials^42^ or the atomic cluster expansion.^43^ Next to density convolutions proposed by Lei and Medford,^41^ descriptors similar to ours have been used also in several other recent works, see refs (44−47). A key distinction is that some of these works use density fitting to construct descriptors for the electronic density. As pointed out by Chen et al.,^48^ the explicit dependence on chemical species makes those ML-DFT functionals less universal.

### 2.2 Machine Learning Scheme

The exchange-correlation energy for any GGA functional can be written as
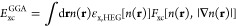
6where ε_x,HEG_ is the exchange energy density for the homogeneous electron gas of uniform density *n*, and *F*_xc_ is the enhancement factor. We use the same form as an ansatz for our ML-RPA model,
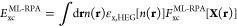
7where **X** is a supervector containing the two- and three-body descriptors. The map **X**(**r**) → *F*_xc_^ML-RPA^(**r**) is found by kernel regression using a Gaussian kernel,
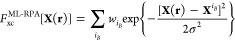
8Here, the kernel width σ is an ML hyperparameter, and the **X**^*i*_*B*_^ are representative kernel control points chosen from the training data. The corresponding weights *w*_*i*_*B*__ are found by solving a linear regression problem. That is, the ML-RPA exchange-correlation energies and ML-RPA exchange-correlation potentials have to agree with RPA reference data in a least-squares sense. The ML-RPA exchange-correlation potentials are obtained by inserting the ansatz (eq 7) into eq 1 and applying the chain rule, see Supplementary Sec. S2.

**Figure 1:**
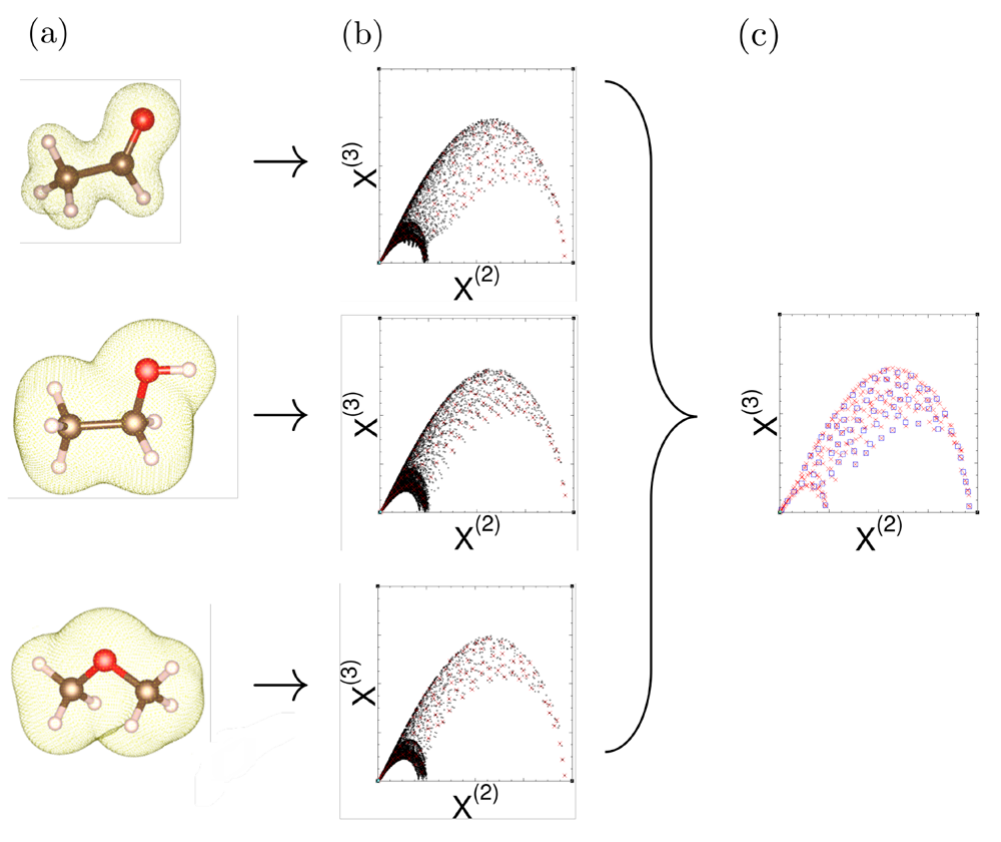
Data sparsification scheme for a training set consisting of three molecules, depicted in column (a). First, using the electronic density as input, we calculate the two-body descriptors *X*_*n*_^(2)^(**r**) and three-body descriptors *X*_*n*_^(3)^(**r**) at each real-space grid point [black dots in column (b)]. A single radial basis function is used here for visualization purposes. Next, a metric is introduced via the Gaussian kernel, and representative density environments (red crosses) are chosen via k-means clustering for each training structure separately. The chosen points are concatenated and a second k-means layer selects the kernel control points **X**^*i*_*B*_^ used in eq 8 [blue squares in column (c)]. For more details, see Supplementary Sec. S2.

**Figure 2:**
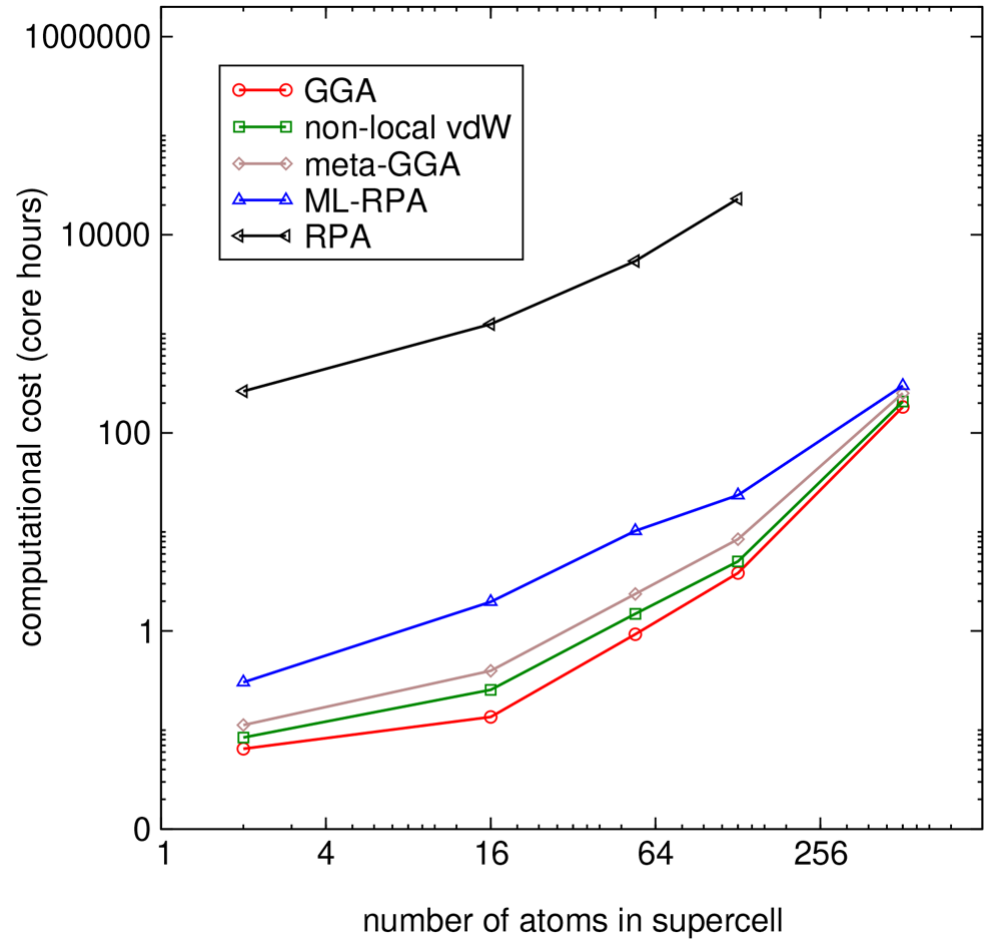
Computational cost of diamond phonon calculations as a function of supercell size; note the log–log scale. For very large system sizes, the computational cost of ML-RPA is dominated by the solution of the Kohn–Sham equation, which scales as . Thus, the computational overhead of ML-RPA calculations with respect to GGA calculations vanishes for very large *N*. Computational details are given in Sec. 3.3.

The exchange-correlation potentials provide derivative information for the ML fit as atomic forces do in MLFFs, thus improving the data efficiency. That is, instead of a single data point per structure for *E*_xc_^RPA^, we have additional data for *v*_xc_^RPA^(**r**) on typically  grid points **r**. As this large amount of data cannot be handled by kernel methods, we must employ data sparsification tools. Without loss of accuracy, the training data can be compressed dramatically by combining k-means clustering^49^ with the metric induced by the Gaussian kernels (see Supplementary Sec. S2). This is illustrated in Figure 1 using a single radial basis function such that the descriptors can be easily visualized in 2D. Once training is complete, the computational cost of evaluating ML-RPA depends linearly on the number of kernel control points used. Further, efficient evaluation of the ML-RPA functional is achieved by extensive use of fast Fourier transforms. We find that ML-RPA is slower than standard GGA functionals by some prefactor for typical applications, but ML-RPA scales as  with system size *N* like GGA does.^50^ In comparison, nonlocal vdW functionals can have the same system size scaling and similar computational cost as ML-RPA,^51^ whereas the RPA is several orders of magnitude slower and has at least  scaling.^52,53^ To give a concrete example, Figure 2 shows the computational cost of diamond phonon calculations as a function of the supercell size. However, we want to stress that relative timings depend also on the concrete system and computational setup.

## 3 Methods

### 3.1 Random-Phase Approximation

As the RPA includes information from unoccupied orbitals as ingredients, it sits on the fifth (highest) rung of Jacob’s ladder. In the last two decades, the RPA has been successfully employed for a multitude of problems, see refs (54) and (55) for reviews. In particular, the RPA is considered a “gold standard” for first-principles surface studies^56−58^ due to its seamless inclusion of vdW interactions next to the good description of covalent and metallic bonds. Combining exact exchange and a good description of dispersion interactions makes the RPA also suitable for water and ice.^59,60^ The usual expression for the RPA exchange-correlation energy reads^61^
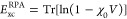
9where χ_0_ is the Kohn–Sham response function, *V* is the Coulomb kernel, and we have used a symbolic notation for sake of brevity. As in standard Kohn–Sham DFT, one can obtain the exchange-correlation potential corresponding to the RPA exchange-correlation energy by taking the functional derivative with respect to the electronic density,
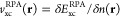
10Plugging in eq 9 and using the chain rule, one can derive the so-called optimized effective potential (OEP) equation,^62−65^ symbolically
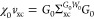
11where *G*_0_ is the noninteracting (Kohn–Sham) Greens-function and Σ_xc_^*G*_0_W_0_^ is the self-energy in the *G*_0_W_0_ approximation. For more details on the implementations of the RPA exchange-correlation energies and the RPA-OEP method, we refer to refs (53) and (37), respectively.

### 3.2 Electronic Self-Consistency

Even tough the RPA-OEP method allows in principle to perform RPA calculations self-consistently,^37^ this procedure is seldom carried out due to the large computational overhead. A more common approach is to evaluate the RPA using orbitals from a semilocal DFT base functional (“RPA on-top of DFT”, RPA@DFT).^67^ Here, we calculate all RPA reference data using PBE as base functional (Perdew–Burke–Ernzerhof, a popular GGA functional^10^). That is, *RPA@PBE is the ground truth for ML-RPA*, and all RPA calculations and ML-RPA calculations are thus performed nonself-consistently on-top of PBE orbitals (unless stated otherwise). A ML substitute functional that reproduces only RPA@PBE would already be very useful; we reiterate that RPA@PBE is standard practice.

**Figure 3:**
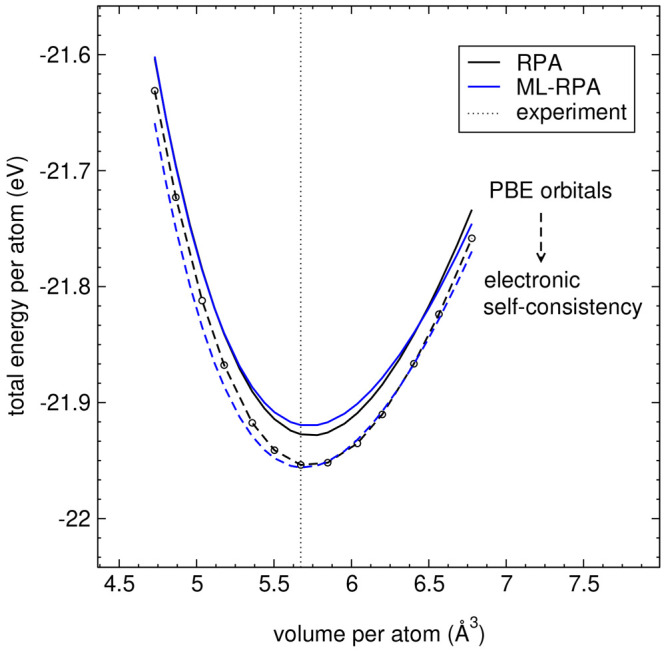
Energy-volume curves of bulk diamond obtained using the RPA and ML-RPA. Full lines indicate calculations on-top of PBE orbitals, and dashed lines indicate self-consistent calculations. Self-consistent RPA reference energies are obtained using the RPA-OEP method. The dotted vertical line indicates the experimental equilibrium volume.^66^

For the calculation of atomic forces, however, it is convenient to perform self-consistent calculations to avoid tedious non-Hellmann–Feynman terms. Wherever RPA reference forces are required, for instance for phonon calculations, we calculate such non-Hellmann–Feynman terms explicitly.^68^ In contrast, ML-RPA can be simply run self-consistently like any semilocal density functional since the exchange-correlation potential is readily available. As a validation, we used the computationally demanding RPA-OEP method to calculate the equilibrium volume of bulk diamond self-consistently (the computational details are given in Sec. 3.3). Figure 3 shows that ML-RPA reproduces the RPA@PBE ground truth well (solid lines). Further, ML-RPA correctly predicts a small downward shift due to electronic self-consistency (dashed lines).

We emphasize that this result is not obvious, as the training set covers only PBE densities. To this point, Snyder et al.^26^ have reported early on that electronic self-consistency can grossly deteriorate the performance of ML-DFT functionals, as self-consistency leads the functional away from its training manifold. Likewise, we observed that earlier versions of ML-RPA became inaccurate when applied self-consistently. However, we find that the current ML-RPA is very stable and reliably converges to a tight energy threshold of 10^–8^ eV. Key hyperparameters in this regard are the cutoff radius (*R*_cut_ = 1.5 Å) and the Tikhonov regularization parameter (*t*_SVD_ = 1.0 × 10^–9^, see Supplementary Sec. S2). Even though atomic densities can be used as starting points, tough preconverging with PBE typically speeds up the ML-RPA self-consistency cycle. Lastly, the ML-RPA stress tensor has also been implemented via finite differences, which is useful for example for volume relaxations and the training of machine learned force fields (see Supplementary Sec. S4).

### 3.3 Computational Details

We use the PAW code VASP (Vienna *Ab Initio* Simulation Package),^69^ adopting the C_GW, H_GW, and O_GW pseudopotentials. All DFT and RPA calculations are performed spin-nonpolarized. An energy cutoff of 600 eV is used for the plane-wave orbital basis set (ENCUT in VASP). For RPA calculations, a reduced cutoff of 400 eV is used to expand the response function χ_0_ (ENCUTGW in VASP), using a cosine window to smoothen the cutoff of the Coulomb kernel.^67,70^ Basis set incompleteness errors are discussed in Supplementary Sec. S3. The one-center PAW contributions to the RPA exchange-correlation energy are treated on the level of Hartree–Fock, consistently for RPA and ML-RPA calculations. The good agreement of *total energies* in Figure 3, rather than relative energies only, demonstrates the consistency of the ML-RPA implementation. Similar agreement is observed for all materials, with fit errors around 1 meV/electron.

## 4 Results

We apply our ML scheme to create an RPA substitute functional for diamond and liquid water. It is important to point out that we create a single ML-RPA functional rather than two separated ones that work only for diamond or water separately.

### 4.1 ML-RPA Training Set

**Figure 4:**
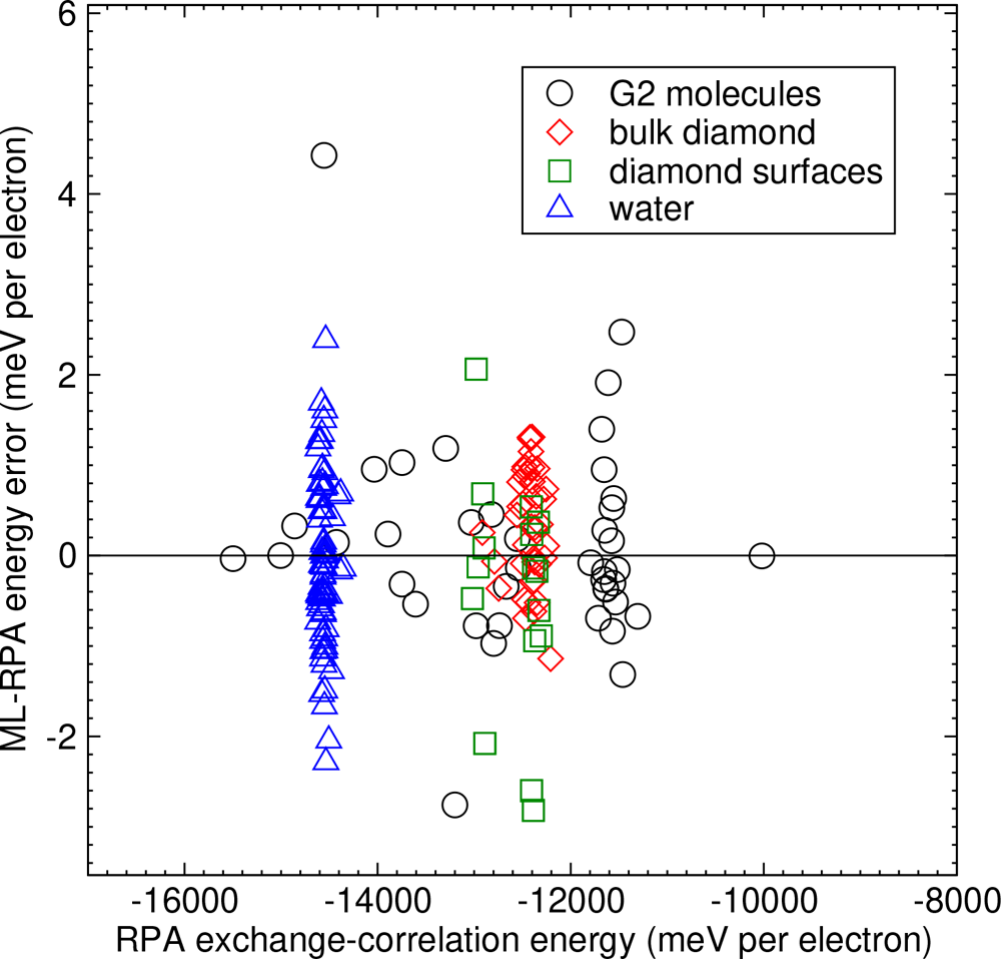
ML-RPA energy fit error, *E*_xc_^ML-RPA^/*N*_*e*_ – *E*_xc_^RPA^/*N*_*e*_. Symbols distinguish between different subgroups of the training set. The H_2_O monomer in the experimental geometry is listed as a G2 molecule.

As a baseline, the ML-RPA training set contains 41 small molecules from the G2-2 database.^71^ We include all nonspin-polarized molecules containing elements C, O, and H except for CH_2_, which does not possess a complete octet structure. We use experimental geometries where available, and otherwise geometries from accurate quantum chemistry calculations.^72^ The molecules can be further classified as 20 hydrocarbons, 19 oxygen substituted hydrocarbons, and 2 inorganic molecules (H_2_ and O_3_). Thus, by including the G2 molecules, we sample basic bonding motives and vacuum regions. To train ML-RPA for our specific applications, we supplement the training set with diamond and water structures. Following a common MLFFs practice,^38^ bulk diamond and bulk water training data are iteratively added from molecular dynamics (MD) simulations, using prior versions of the ML-RPA functional to create the MD trajectories. For bulk diamond, we pick 20 MD snapshots from the 8 atom supercell and 20 snapshots from the 16 atom supercell. For bulk water, we add 32 liquid water structures containing 8 water molecules to the ML-RPA training set as well as 39 structures using larger supercells (31 or 32 water molecules). In addition, the ML-RPA training set contains 6 structures sampling the water monomer (this includes the water monomer at the experimental geometry, which is a G2 molecule). Finally, we added 16 diamond surfaces with different surface terminations to the training set. To compensate for the small amount of surface data, these 16 surfaces are included twice in the training set. The training set surfaces and 12 other surfaces left for tests are characterized in detail in Supplementary Sec. S3. Figure 4 details the ML-RPA fit errors for exchange-correlation energies (exchange-correlation potentials are discussed in Supplementary Sec. S2.). The data spread along the *y*-axis indicates that ML-RPA balances the fit well across the different subgroups. Further, the spread along the *x*-axis shows that the G2 molecules contain very different chemical environments, whereas the other training data are more homogeneous.

### 4.2 Bulk Diamond

**Figure 5:**
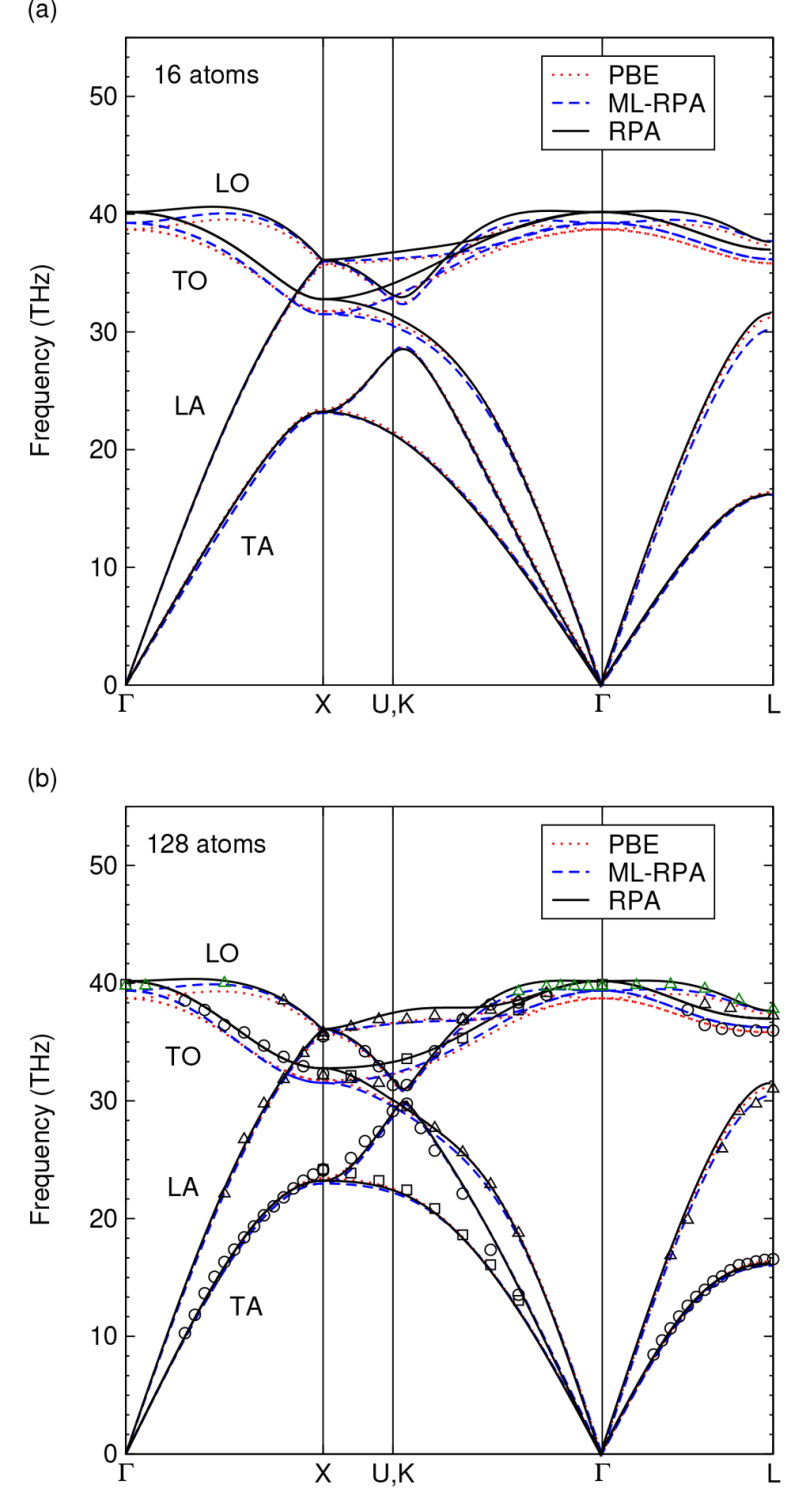
Phonon dispersion of diamond, calculated at the respective equilibrium lattice constants, using supercells containing (a) 16 and (b) 128 atoms. Phonons obtained using the RPA are represented as solid black lines, blue dashed lines represent ML-RPA. PBE calculations are also shown for comparison (red dotted lines). ML-RPA has training data only for the smaller supercell size [16 atoms, panel (a)]. Black and green symbols indicate experimental data from refs (66) and (75), respectively.

In the following, we calculate phonon dispersions using the finite displacement method.^73,74^ Converged results are obtained in the large supercell limit, which is hard to achieve with RPA due to its unfavorable scaling behavior. Figure 5 compares the ML-RPA phonon dispersions for different supercell sizes to the RPA results. For contrast, phonon dispersions obtained using PBE are shown as well. For the 16 atom supercell [panel (a)], where training data are available, the ML-RPA phonon dispersion is generally in good agreement with the RPA, though high-frequency modes are slightly underestimated. The RPA calculation for the larger 128 atom supercells validates the extrapolation ability of ML-RPA, as no training data are included for this supercell size. A prominent finite size effect is the closing of the gap near the K-point with respect to the smaller 16 atom cell. Further, the overbending of the LO modes reduces with increasing supercell size, which is most notable along Δ (Γ-X). These characteristic features are well reproduced with ML-RPA. The phonon dispersions obtained using the RPA are overall in good agreement with experimental data,^66,75^ whereas ML-RPA and PBE slightly underestimate the high-frequency modes.

### 4.3 Diamond Surfaces

**Figure 6:**
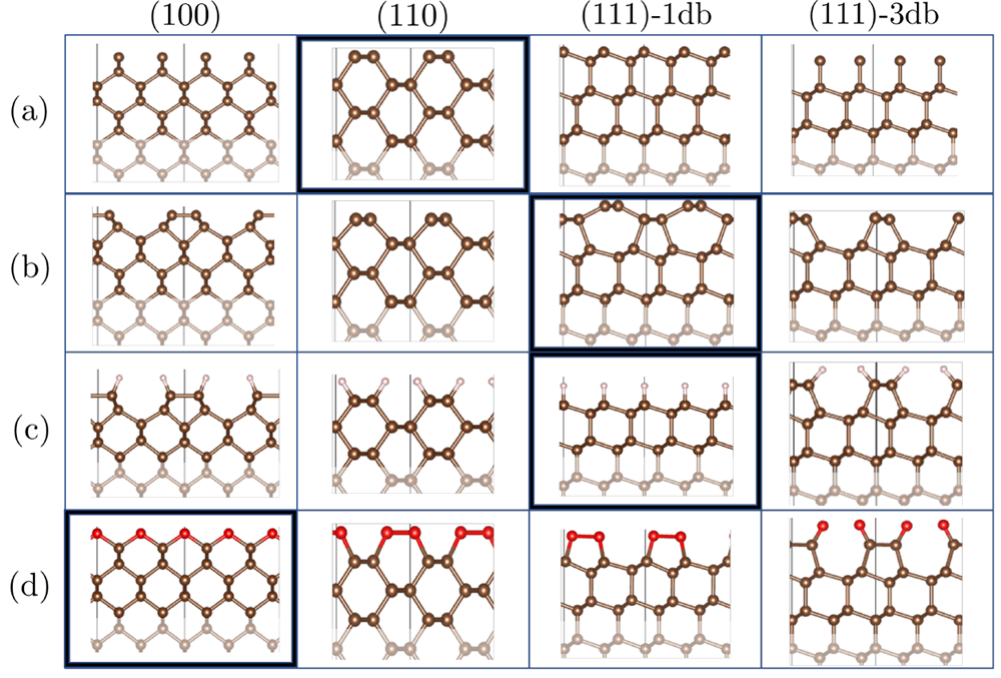
Side view of crystallographic diamond surfaces.^76^ The 2 × 1 simulation cells are indicated by black vertical lines, and bulk regions are indicated by faded atoms. From top to bottom: (a) clean surfaces, (b) reconstructed surfaces, (c) hydrogenated surfaces (1 ML), and (d) oxygenated surfaces (1 ML). Bold edges indicate the most stable orientation for a given surface termination as predicted by the RPA. Whereas the (111)-1db surface dereconstructs upon the chemisorption of hydrogen and oxygen,^77^ the (100) surface dereconstructs only upon the chemisorption of oxygen.^78^ The (111)-3db surface retains the 2 × 1 geometry throughout,^79^ and the (110) surface shows no 2 × 1 reconstruction at all.^80^

The advent of chemical vapor deposition (CVD) has encouraged detailed first-principles simulations of diamond surfaces. Specifically, the characterization of ideal crystallographic surfaces has proven useful for the theoretical understanding of CVD grown diamond.^81^ When a diamond surface is cut, the outer surface layers can rearrange to partially eliminate the exposed dangling bonds; see Figure 6. These reconstructions significantly change the properties of the surfaces. Further, important material properties such as electron affinity can be modified via chemisorption processes in a controlled fashion, the most important surface adsorbates being H and O. The (100) surface is the most relevant crystallographic surface and has been thoroughly studied.^78,82^ While the (111) surface has also received a lot of attention,^77,83^ most studies neglect that there are two possible ways to cut this surface.^79,84^ Namely, via the so-called glide and shuffle planes, one can expose (111) diamond surfaces with one dangling bond (1db) or three dangling bonds (3db) per surface atom, respectively. Even though the clean 1db surface is clearly more stable, 3db surfaces naturally occur during growth and etching processes. Finally, the (110) surface, which is difficult to prepare experimentally, has only recently been fully characterized.^85^ In the following, we calculate diamond surface energies of formation via
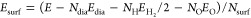
12where *E* is the total energy of the surface, *E*_dia_ is the energy per atom of bulk diamond, *E*_H_2__ is the energy of the H_2_ molecule, and *E*_O_ is calculated using the water monomer as reference
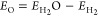
13Vibrational contributions to the formation energies due to zero-point motion are neglected. Diamond surface calculations are performed in 2 × 1 supercells using symmetric slabs. We used 18 surface layers for the (111)-1db surface and 16 layers for the rest. This assures convergence of the surface formation energies to better than 10 meV accuracy.^80,83^ The surface geometries were obtained using the PBE functional; further details are given in Supplementary Sec. S4. RPA formation energies of diamond surfaces are collected in Table 1. The (100) and (111)-1db surfaces were included in training, whereas the (110) and (111)-3db surfaces are left as independent tests for ML-RPA. For all surface terminations studied, ML-RPA correctly predicts the most stable surface orientation (underlined values). For example, of the clean surfaces (as cut), both RPA and ML-RPA predict the order (110) < (111)-1db < (100) < (111)-3db, which can be qualitatively understood via coordination of the surface atoms.^83^ Note that the (111)-3db surface becomes increasingly competitive with the (111)-1db surface as more dangling bonds are eliminated via reconstruction (-1 db) and chemisorption (-2 db for H and -3 db for O).

**Table 1:** RPA Energies of Formation for Diamond Surfaces, Calculated via Eq. 12 (in eV per Surface Atom)^a^

	(100)^b^		(110)		(111)-1db^b^		(111)-3db	
	RPA	ML-RPA	RPA	ML-RPA	RPA	ML-RPA	RPA	ML-RPA
clean	3.66	3.57	2.09	1.95	2.69	2.52	4.37	4.37
reconstructed	2.08	2.03	1.70	1.59	1.41	1.34	2.70	2.56
+H (1 ML)	0.20	0.11	–0.11	–0.12	–0.19	–0.24	0.17	0.11
+O (1 ML)	1.99	1.91	3.23	3.21	2.99	2.94	2.90	2.79

a The most stable surface orientation for a given surface termination is underlined, and surfaces included in the ML-RPA training set are marked by asterisks. Surface calculations are performed in 2 × 1 supercells, with geometries illustrated in Figure 6.

b included in the ML-RPA training set.

Including calculations of metastable surfaces (see Supplementary Sec. S3), we have assembled a database of 28 RPA surface formation energies in total. It is interesting to use these data as benchmark for other exchange-correlation functionals. Table 2 shows that all functionals, including ML-RPA tend to predict smaller formation energies than the RPA (negative mean relative errors). In terms of accuracy, ML-RPA performs very well, with a mean absolute error of 70 meV per surface atom. Surface energies predicted by the meta-GGA functional SCAN as well as the vdW functionals PBE+TS and SCAN+rVV10 are also in very good agreement with the RPA, with mean absolute errors slightly larger than ML-RPA. Finally, we comment again on the stability of electronic self-consistency. Going from ML-RPA@PBE to self-consistent ML-RPA, individual surface energies change by 60 meV per surface atom or less, and the mean absolute error of self-consistent ML-RPA (compared to RPA@PBE) is only 80 meV per surface atom. Thus, the accuracy of ML-RPA is not significantly diminished by electronic self-consistency.

**Table 2:** Surface Formation Energies from Different Exchange-Correlation Functionals Compared to Values from the RPA^a^

	MSE	MAE	MAX
LDA	–0.06	0.14	0.34
PBEsol	–0.10	0.12	0.33
PBE	–0.19	0.19	0.39
RPBE	–0.23	0.24	0.44
PBE+TS	–0.01	0.08	0.25
RPBE+D3(BJ)	–0.09	0.12	0.48
optB88-vdW	–0.19	0.19	0.62
rev-vdW-DF2	–0.13	0.14	0.52
rVV10	–0.18	0.18	0.57
SCAN	–0.08	0.11	0.25
SCAN+rVV10	–0.03	0.10	0.24
ML-RPA	–0.07	0.07	0.18

a Mean signed error (MSE), mean absolute error (MAE), and maximum absolute error (MAX) are given in eV per surface atom. Averages are over 28 diamond surfaces, of which 16 are in the ML-RPA training set; see Supplementary Sec. S3.

### 4.4 Liquid Water

Water with its many anomalies is both an important and challenging system for first-principles molecular dynamics simulations.^86^ The role of the exchange-correlation functional for the description of liquid water and ice has been studied extensively.^59,87−89^ This has made water an interesting target for several recent MLFF^60,90,91^ and ML-DFT^22,45^ approaches. In particular, Yao and Kanai^60^ used MLFFs to perform RPA-level calculations for liquid water with the inclusion of nuclear quantum effects. They showed that the RPA can well reproduce experimental data for numerous water properties at different temperatures. Due to the small mass of the hydrogen atom, the oxygen–hydrogen radial distribution function (RDF) and especially the hydrogen–hydrogen RDF are significantly altered by nuclear quantum effects. Namely, classical MD predicts an oxygen–hydrogen RDF that is overstructured compared to experimental data and even more so for the hydrogen–hydrogen RDF. In contrast, the oxygen–oxygen RDF is far less affected, especially for higher temperatures.

Accurate determination of the water RDFs requires long MD simulations that are computationally expensive even without nuclear quantum corrections. Thus, we perform classical MDs using 64 water molecules and speed them up by combining ML-RPA with MLFFs. That is, we train a machine learning force field “on-the-fly”^32,92^ using the energies and forces predicted by ML-RPA. In order to obtain an RPA reference for the radial distribution function, we also train an MLFF directly on RPA energies and forces (RPA-MLFF). The RPA-MLFF training set contains all water structures from the ML-RPA training set, plus 30 additional structures containing 32 molecules. To validate our machine learning force fields, we also trained on-the-fly MLFFs for the vdW functionals RPBE+D3(BJ)^93,94^ and PBE+TS.^95^ Further details of the MLFF setups are given in Supplementary Sec. S4.

**Figure 7:**
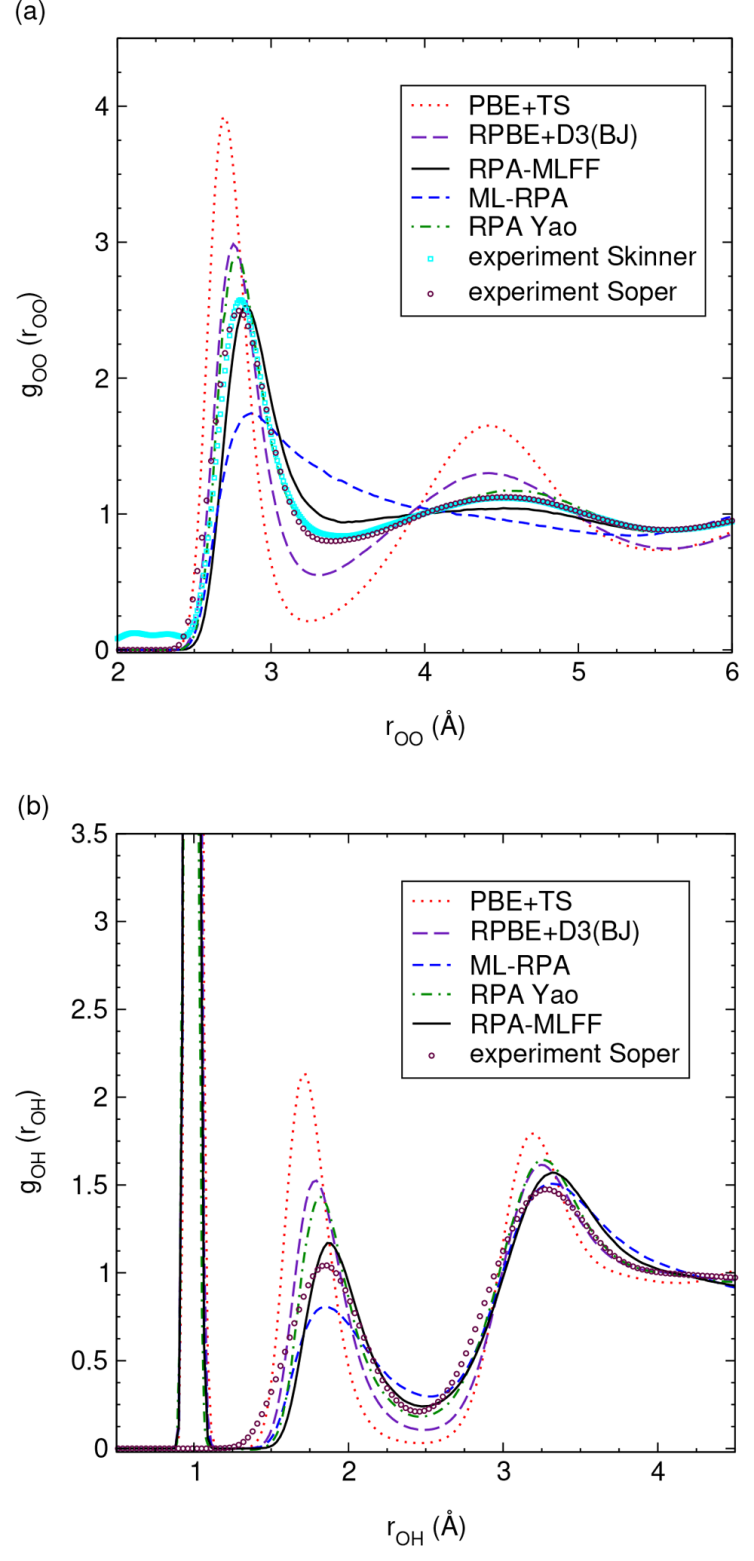
Partial radial distribution functions of liquid water at ambient conditions (*T* = 300 K, ρ = 1 g/cm^3^), details of the MD simulations are given in Supplementary Sec. S4. (a) Oxygen–oxygen radial distribution function and (b) oxygen–hydrogen radial distribution function. RPA results from Yao and Kanai are extracted from ref (60); experimental data are taken from refs (96, 97). See the text for a discussion of nuclear quantum effects.

Figure 7 shows the oxygen–oxygen RDF, *g*_OO_, and oxygen–hydrogen RDF, *g*_OH_, under ambient conditions. First, we note that the RDFs obtained from PBE+TS and RPBE+D3(BJ) are in good agreement with respective literature results^98,99^ (for PBE+TS, we compared the RDF at *T* = 330 K, not shown). The PBE+TS RDF is clearly overstructured compared with experimental data. The water structure is “too tetragonal”, even more so when the TS vdW correction is not included, see ref (98). The RPBE+D3(BJ) RDF is in better agreement with experiment but is still somewhat overstructured. This is a specific effect of the Becke–Johnson (BJ) damping, that is, the RPBE+D3(0) RDF (using zero damping) is closer to experiment (see ref (99)). Next, the RDFs predicted by RPA-MLFF are less structured than the RPA references of Yao and Kanai, comparing the first peaks and minima of both *g*_OO_ and *g*_OH_. This discrepancy is possibly due to technical convergence of either RPA calculation, in particular, basis set incompleteness errors (see Supplementary Sec. 4 for further discussion). The fact that RPA-MLFF closely reproduces the first experimental peak height of *g*_OO_ is arguably accidental since nuclear quantum effects are not included. For *g*_OH_, however, the result of Yao and Kanai is still more structured than experiment even with nuclear quantum effects included (see Figure 1 in ref (60).), indicating that our RPA reference is potentially more accurate.

That said, overall, both RPA reference results agree well with experimental data. Turning to the water structure predicted by ML-RPA, there are some clear discrepancies with respect to both RPA references. The first peak of *g*_OO_ is smaller in height, and the second peak is completely missing. The *g*_OH_ predicted by ML-RPA is also somewhat less structured than the RPA references, but the overall agreement is better than that for *g*_OO_. Importantly, the discrepancies are mainly due to the actual ML-RPA density functional since the on-the-fly MLFF is very accurate (see Supplementary Sec. S4). Specifically, atomic forces for all MLFFs trained here exhibit a root-mean-square error of roughly 30 meV Å^–1^, whereas the respective force errors for liquid water due to ML-RPA are roughly 90 meV Å^–1^. It is also important to point out that the RPA-MLFF is a single-purpose force field, trained specifically to simulate liquid water in the bulk (and the water monomer). In contrast, the ML-RPA functional is not limited in this way as will be demonstrated in the following section.

### 4.5 Smaller Water Clusters

**Figure 8:**
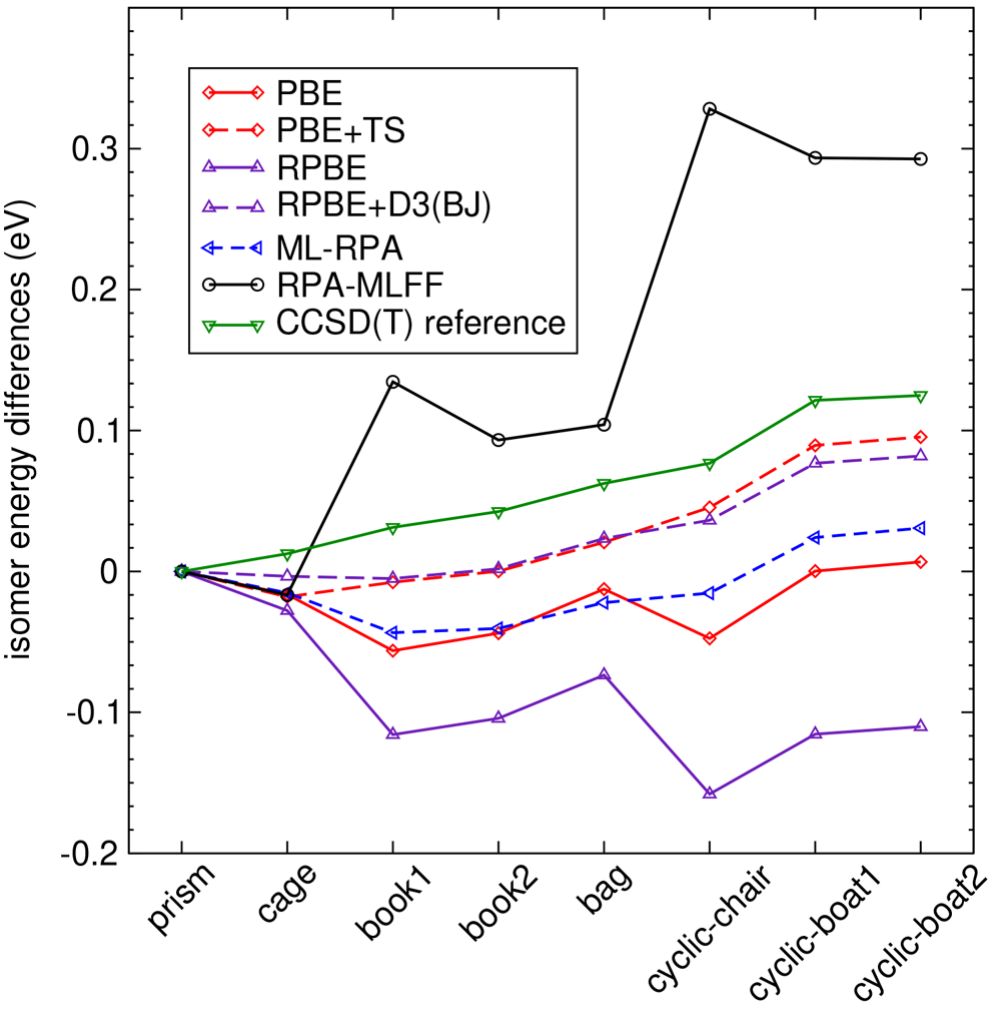
Binding energy differences of eight H_2_O hexamers. Geometries and CCSD(T) reference data are taken from ref (101). The vdW functionals PS+TS and RPBE+D3(BJ) largely correct the errors of their respective GGA base functionals; see also Supplementary Sec. S6. Lines drawn are only guides to the eye.

An important H_2_O benchmark is the performance for smaller water clusters.^88^ Already the simple H_2_O dimer gives a predictive measure of the strength of a hydrogen bond in liquid water. Table 3 shows that ML-RPA predicts a somewhat underbound dimer, that is the binding energy is too small and the bond lengths are too small compared to the accurate quantum chemistry reference.^100^ This underbinding behavior of ML-RPA is consistent with that of the understructured liquid. Going to larger clusters, the water hexamers present a difficult challenge for DFT, as three-dimensional structures (“prism”, “cage”, “bag”) compete energetically with two-dimensional ones (“book”, “chair”, “boat”). Figure 8 shows that ML-RPA erroneously predicts two-dimensional structures to be most stable, as do all GGA functionals (see also Supplementary Sec. S5). LDA and the SCAN meta-GGA functional perform well in this regard but overbind the dimer, see Table 3. All vdW functionals tested give excellent results for both the dimer as well as the hexamers. This confirms the critical role that vdW interactions play for the structure of water.^88,90^ Further, RPA-MLFF clearly fails for water clusters, but we reiterate that it has been trained only for liquid water in the bulk. The fact that the RPA-MLFF dimer binding energy is close to the accurate quantum chemistry reference is likely fortuitous since the dimer bond lengths are much too large. The hexamer binding energies are also quite erratic for RPA-MLFF. The fact that RPA-MLFF extrapolates is substantiated by large Bayesian error predictions: compared to typical bulk water configurations, the maximum force Bayesian error is three times as large for the hexamers and five times as large for the dimer.

**Table 3:** Ground State Properties of the H_2_O Dimer^a^

	*E*_b_^dim^	*d*_OO_^dim^	*d*_OH_^dim^
LDA	0.39	2.72	1.73
PBEsol	0.27	2.80	1.82
PBE	0.23	2.88	1.91
RPBE	0.17	3.02	2.04
PBE+TS	0.24	2.89	1.92
RPBE+D3(BJ)	0.21	2.94	1.97
optB88-vdW	0.22	2.90	1.93
rev-vdW-DF2	0.23	2.89	1.92
rVV10	0.25	2.88	1.90
SCAN	0.25	2.84	1.88
SCAN+rVV10	0.26	2.84	1.88
ML-RPA	0.18	3.07	2.10
RPA-MLFF	0.21	3.28	2.30
CCSD(T)^100^	0.22	2.91	1.96

a Dimer binding energies, *E*_b_^dim^ = −*E*[(H_2_O)_2_] + 2*E*[H_2_O], are given in eV. Equilibrium bond lengths *d*_OO_^dim^ and *d*_OH_^dim^ are given in Å. All calculations are performed fully self-consistently.

Finally, we investigated cubic ice, specifically, the I_c_(a) proton-ordered ice phase as described in ref (59). The equilibrium volume predicted by RPA-MLFF is 32.3 Å^3^ per H_2_O, in good agreement with the 32.6 Å^3^ per H_2_O obtained using RPA. The ML-RPA equilibrium volume is somewhat smaller (31.2 Å^3^ per H_2_O). For comparison, the equilibrium volumes predicted by PBE and PBE+TS are 30.2 Å^3^ per H_2_O and 30.0 Å^3^ per H_2_O, respectively. In summary, ML-RPA provides a consistent but somewhat inaccurate description of water and ice. The fact that ML-RPA misses the second *g*_OO_ maximum, underbinds the water dimer, and provides a PBE-like description of the water hexamers and cubic ice strongly indicates that the current ML-RPA misses some crucial nonlocal interactions. This is likely connected to the rather small cutoff radius used here for ML-RPA (*R*_cut_ = 1.5 Å). However, increasing the cutoff is not beneficial for the current ML-RPA, and our tests indicate that this diminishes ML-RPA accuracy (see Supplementary Sec. S2).

### 4.6 Homogeneous Electron Gas

**Figure 9:**
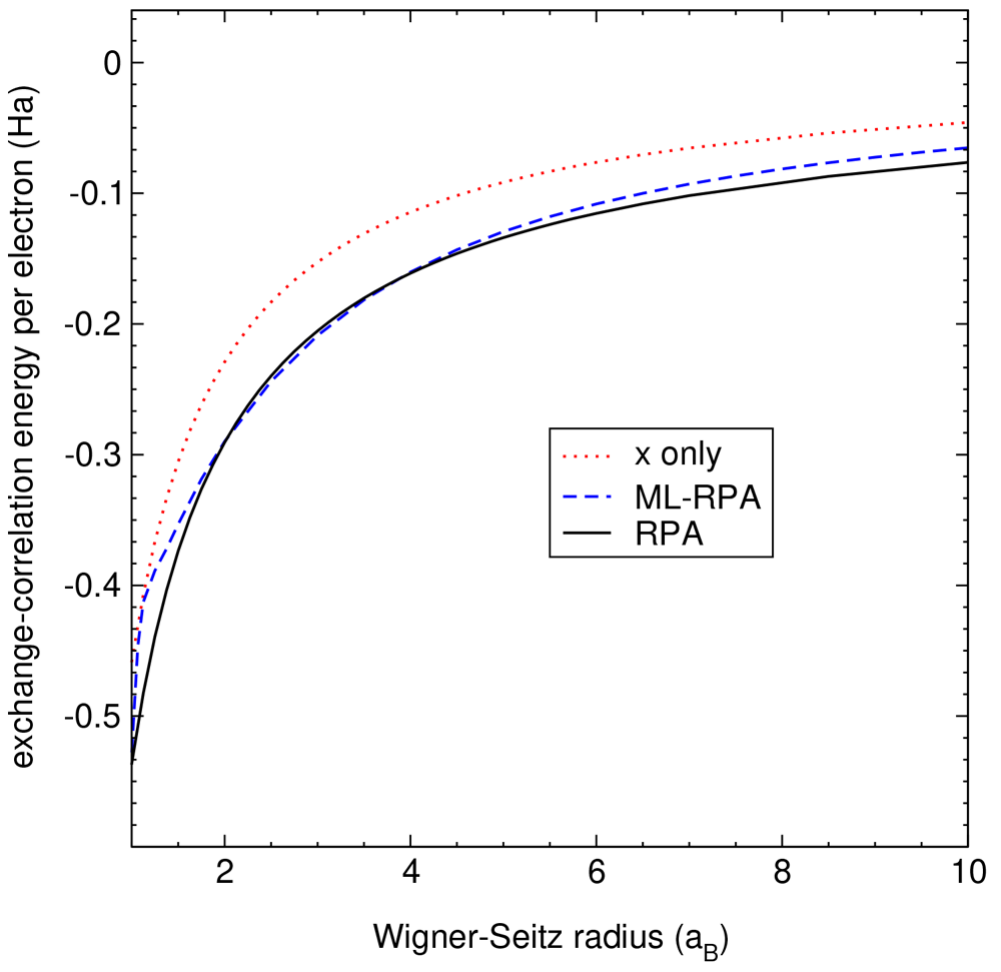
Application of ML-RPA to the homogeneous electron gas. RPA results were taken from ref (70); the exchange-only approximation ε_x, HEG_ is also shown for comparison. No HEG data were added to the ML-RPA training set.

To challenge the extrapolation abilities of our ML-RPA functional, we apply it to the homogeneous electron gas (HEG). The HEG constitutes an “appropriate norm”, which is an important theoretical limit that density functionals should fulfill. Due to symmetry, the HEG is completely characterized by the electron density *n*, or equivalently the Wigner–Seitz radius *r*_s_ = (3/4*πn*)^1/3^. Starting with LDA, many successful exchange-correlation functionals are designed such that they describe the HEG exactly, but this is not the case for the present ML-RPA functional. It is important to point out that the ground truth here is the exchange-correlation energy per electron as given by the RPA. As the RPA is itself an approximation, ε_xc,HEG_^RPA^ differs from the usual LDA, which is based on exact numerical data from Quantum Monte Carlo calculations.^102^Figure 9 shows that ML-RPA for intermediate densities (*r*_s_ ∼ 2–5) closely follows the RPA reference. Since this is the range of physical densities, ML-RPA seems to have learned the HEG indirectly from diamond and water data. Still, this excellent agreement is somewhat surprising considering that no HEG data were explicitly added to the ML-RPA training set. Furthermore, for small densities (*r*_s_ ≳ 5), ML-RPA is slightly less accurate but still well behaved. Finally, for large densities (*r*_s_ ≲ 2), ML-RPA develops an unphysical kink. This indicates the presence of an “extrapolation hole”, that is, the complete lack of training data can cause erratic behavior.

## 5 Discussion

The LDA and different GGA functionals differ in the strengths of their respective enhancement factors. This results, for example, in the following trend for cohesive energies of solids
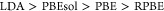
14The same trend is also manifest for surface formation energies and molecular adsorption energies.^56^ As different enhancement strengths fit better for different physical properties, this leads to a well-known trade-off for GGA functionals. This trade-off is visible also in the current study: LDA and PBEsol give better diamond surface energies (Table 2). PBE and RPBE perform better for the water dimer (Table 3), but the trend is reversed for the hexamer puzzle (Supplementary Sec. S5). This means that no GGA functional can give a completely satisfactory description of liquid water and ice (see also ref (88) for a more in-depth discussion). We have demonstrated that the nonlocal gradient approximation used in our ML-RPA model can overcome the GGA trade-off to some extend. While ML-RPA does not exceed a GGA-level description of liquid water, it clearly outperforms all GGA functionals for the diamond surface benchmark.

Traditional routes beyond GGA are metaGGA and hybrid functionals on the one hand and vdW functionals on the other hand. The RPA itself can be considered as the “gold standard” of vdW functionals.^103^ Different vdW functionals tested here generally outperform their respective semilocal DFT base functionals, though the improvement is not always consistent. For example, the rVV10 vdW correction slightly increases the surface energies of the pristine SCAN functional, achieving good agreement with the RPA (Table 2, ref (57)). On the other hand, SCAN already slightly overbinds the water dimer and gives a somewhat overstructured liquid. Thus, it is plausible that additional binding in the form of rVV10 can only deteriorate the performance of SCAN for water (Supplementary Sec. S5, ref (104)). We reason that the rather small cutoff radius adapted here for ML-RPA is not sufficient to capture the full nonlocality that is required for a complete description of liquid water (ML-RPA uses a cutoff radius of 1.5 Å, whereas RPA-MLFF uses a larger cutoff of 6.0 Å). However, simply increasing the cutoff radius is not an option with the current implementation and training database. We repeat that our tests show that a larger cutoff would diminish the fit accuracy. Future work could instead focus on the construction of explicitly long-range descriptors as present in nonlocal vdW functionals or in recent extensions of MLFF frameworks.^105^

Finally, a common problem of ML techniques is extrapolation; that is, one can only expect good performance if applications are similar enough to the respective training sets. Here, this point was demonstrated by the poor performance of RPA-MLFF for water clusters. (RPA-MLFF has been trained only for bulk water and the water monomer.) In contrast, ML-RPA gives consistent, if inaccurate, predictions, though it has less water training data. We speculate that this is due to ML-RPA descriptors being much more compact, which makes extrapolation more manageable. Specifically, here we used 8 density descriptors for ML-RPA versus 408 atomic descriptors for RPA-MLFF (204 for both O and H). It is worth pointing out that this difference will be exacerbated when more chemical species are described at the same time. That is, the number of descriptors in MLFF schemes generally scales unfavorably with the number of chemical species, whereas the density descriptors used for ML-RPA do not depend explicitly on the atom type. On the other side, RPA-MLFF is in the present implementation undeniably superior in terms of raw fit accuracy. ML-RPA and RPA-MLFF thus strike different balances between the model flexibility and universality.

When pushed even further outside of its training set, however, ML-RPA eventually extrapolates as well as is demonstrated for the homogeneous electron gas. The obvious but costly solution to the extrapolation problem is the construction of ever larger training databases. A more elegant alternative would be to enforce ML-RPA to obey various known exact constraints. Exact constraints have been used in the past to construct successful functionals such as the SCAN functional (“strongly constrained and appropriately normed”).^12^ Recently, there have been some promising efforts to incorporate exact constraints also into machine learned density functionals.^22,47,106,107^

## 6 Conclusion and Outlook

In this work, we have machine learned a substitute density functional based on the random-phase approximation. The ingredients of ML-RPA are density descriptors constructed analogously to the two- and three-body descriptors used for machine learning force fields. These ingredients can be considered as nonlocal extensions of the local density and its gradient. As a first application, we constructed an ML-RPA functional for diamond and liquid water. We have demonstrated how such a functional can be used to enable RPA calculations at a larger scale. For a data set of 28 diamond surfaces, ML-RPA surpasses all tested GGA functionals in terms of accuracy and reaches the level of state-of-the-art vdW functionals. For liquid water, ML-RPA is less accurate and falls back to a GGA-level description, which we traced back to an insufficient description of nonlocal interactions.

Our ML-RPA scheme was demonstrated to learn fairly quickly from small amounts of RPA data, with the entire database consisting of less than 200 structures. We credit this data efficiency to the inclusion of derivative information in the form of RPA exchange-correlation potentials, which are obtained via the optimized effective potential method. This is in close analogy to fitting atomic forces in MLFFs. Generally, the tasks of machine learning atomic force fields and machine learning density functionals are closely related. We hope to see continued exchange of concepts and techniques, as we believe that both fields can benefit immensely from such a “cross-fertilization” of ideas.

Finally, our machine learning method using optimized effective potentials is general and is not limited to the random-phase approximation. For example, beyond RPA theories can be constructed by including vertex corrections to the screened Coulomb interaction and/or self-energy. One can also envision extracting high accuracy exchange-correlation potentials from accurate coupled-cluster densities via Kohn–Sham inversion. Our method can also be applied to learn hybrid functionals, where large databases can be obtained more easily, thus facilitating large-scale hybrid functional simulations.

## Data Availability

The ML-RPA training data is freely available at 10.25365/phaidra.418.

## Supporting Information Available

Analytical proof of Eqs. 3 and (5) (S1), analytical expressions for the ML-RPA exchange-correlation potentials (S2), details of our data sparsification scheme (S2), ML hyperparamters (S2), characterization of the diamond surfaces (S3), training details for machine learned force fields (S4), water hexamer benchmark for more DFT functionals (S5) (PDF)
