# Supplementary material for: Machine Learning Density Functionals from the Random-Phase Approximation
Source: J Chem Theory Comput. 2023 Oct 6;19(20):7287–99. doi: 10.1021/acs.jctc.3c00848 (PMC10601474; doi:10.1021/acs.jctc.3c00848)
Supplement: Supplementary file 1 — ct3c00848_si_001.pdf [file ct3c00848_si_001.pdf]

Supporting Information: Machine Learning Density Functionals from the Random-Phase Approximation

Stefan Riemelmoser,*,†,‡ Carla Verdi,†,¶,§ Merzuk Kaltak,‖ and Georg Kresse†,‖

†*Faculty of Physics and Center for Computational Materials Science, University of Vienna, Kolingasse 14-16, A-1090 Vienna, Austria*

‡*Vienna Doctoral School in Physics, University of Vienna, Boltzmanngasse 5, A-1090 Vienna, Austria*

¶*School of Physics, The University of Sydney, Sydney, New South Wales 2006, Australia*

§ *School of Mathematics and Physics, The University of Queensland, Brisbane, Queensland 4072, Australia*

‖*VASP Software GmbH, Sensengasse 8/12, A-1090 Vienna, Austria*

E-mail: stefan.riemelmoser@univie.ac.at.

S1 Nonlocal density descriptors

In the following, we describe our density descriptors in more detail and comment on how they can be evaluated efficiently via the use of fast Fourier transforms (FFTs). We begin by expanding the electronic density around each grid point $\mathbf{r}$ [Eq. (2) in the main text]

$$n(\mathbf{r}+\mathbf{r}')f_{\text{cut}}(r') = \sum_{nlm} c_{nlm}(\mathbf{r})\phi_{nl}(r')Y_l^m(\widehat{\mathbf{r}'}). \tag{S1}$$

For the cutoff function f_{cut}, we use the cosine cutoff proposed by Behler and Parrinello,[S1]

$$f(r') = \begin{cases} \frac{1}{2}\left[1 + \cos\left(\frac{\pi r'}{R_{\text{cut}}}\right)\right] & 0 \leq r' \leq R_{\text{cut}} \\ 0 & r \geq R_{\text{cut}}. \end{cases} \tag{S2}$$

For radial basis functions ϕ_{nl}, we use spherical Bessel functions as in ref S2,

$$\phi_{nl}(r') = j_l(q_{nl}r'), \tag{S3}$$

where the q_{nl} are chosen such that the basis functions vanish at $r' = R_{\text{cut}}$. Such spherical Bessel functions form a complete basis on the interval $[0, R_{\text{cut}}]$ and fulfill orthogonality relations of the kind

$$\int_0^{R_{\text{cut}}} \mathrm{d}r \; r^2 j_l(q_{nl}r) j_l(q_{n'l}r) = \delta_{nn'} A_{nl}. \tag{S4}$$

For example, in the case of $l = 0$ one obtains

$$\phi_{n0}(r') = j_0(q_{n0}r') = \frac{\sin(q_{n0}r')}{q_{n0}r'}, \tag{S5}$$

and demanding that $\sin(q_{n0}R_{\text{cut}}) = 0$ yields the simple expressions

$$\begin{aligned} q_{n0} &= \frac{n\pi}{R_{\text{cut}}} \\ A_{n0} &= \frac{R_{\text{cut}}^3}{2n^2\pi^2} \qquad n = 1, 2, ... \end{aligned} \tag{S6}$$

Next, we calculate the expansion coefficients c_{nlm} using the orthogonality relations (S4), as well as those for the Y_l^m,

$$\int \mathrm{d}\Omega \; Y_l^m(\hat{\mathbf{r}}) Y_{l'}^{m'}(\hat{\mathbf{r}}) = \delta_{ll'}\delta_{mm'}, \tag{S7}$$

yielding

$$
\begin{aligned}
c_{nlm}(\mathbf{r}) &= \int_0^{R_{\text{cut}}} \mathrm{d}r' r'^2 \frac{\phi_{nl}(r') f_{\text{cut}}(r')}{A_{nl}} \int \mathrm{d}\Omega' Y_l^m(\widehat{\mathbf{r}'}) n(\mathbf{r}+\mathbf{r}') \\
&= \int_0^{R_{\text{cut}}} \mathrm{d}r' r'^2 \tilde{\phi}_{nl}(r') \int \mathrm{d}\Omega' Y_l^m(\widehat{\mathbf{r}'}) n(\mathbf{r}+\mathbf{r}'),
\end{aligned} \tag{S8}
$$

where we have absorbed the coefficients A_{nl} and the cutoff function f_{cut} to define modified basis functions $\tilde{\phi}_{nl}$,

$$
\tilde{\phi}_{nl}(r') = \frac{\phi_{nl}(r') f_{\text{cut}}(r')}{A_{nl}}. \tag{S9}
$$

Next, we combine the Fourier representation of the density

$$
n(\mathbf{r}+\mathbf{r}') = \int \frac{\mathrm{d}\mathbf{q}}{(2\pi)^3}\, e^{i\mathbf{q}(\mathbf{r}+\mathbf{r}')} n(\mathbf{q}), \tag{S10}
$$

and the plane-wave expansion,[S3]

$$
e^{i\mathbf{q}\mathbf{r}'} = 4\pi \sum_{lm} i^l j_l(qr') Y_l^m(\widehat{\mathbf{q}}) Y_l^m(\widehat{\mathbf{r}'}), \tag{S11}
$$

to rewrite Eq. (S8) as

$$
c_{nlm}(\mathbf{r}) = 4\pi \int_0^{R_{\text{cut}}} \mathrm{d}r' r'^2 \tilde{\phi}_{nl}(r') \int \mathrm{d}\Omega' Y_l^m(\widehat{\mathbf{r}'}) \int \frac{\mathrm{d}\mathbf{q}}{(2\pi)^3} e^{i\mathbf{q}\mathbf{r}} n(\mathbf{q}) \sum_{l'm'} i^{l'} j_{l'}(qr') Y_{l'}^{m'}(\widehat{\mathbf{q}}) Y_{l'}^{m'}(\widehat{\mathbf{r}'}). \tag{S12}
$$

To simplify the equation above, we use the orthogonality relations of the Y_l^m [Eq. (S7)] and define the spherical Bessel transform,

$$
\begin{aligned}
\tilde{\phi}_{nl}(q) &= 4\pi \int_0^{R_{\text{cut}}} \mathrm{d}r\ r'^2 j_l(qr') \tilde{\phi}_{ln}(r') \\
\leftrightarrow \tilde{\phi}_{nl}(r') &= \frac{1}{2\pi^2} \int \mathrm{d}q\ q^2 j_l(qr') \tilde{\phi}_{nl}(q),
\end{aligned} \tag{S13}
$$

to finally arrive at the concise expression

$$
c_{nlm}(\mathbf{r}) = i^l \int \frac{\mathrm{d}\mathbf{q}}{(2\pi)^3} e^{i\mathbf{q}\mathbf{r}} n(\mathbf{q}) \tilde{\phi}_{nl}(q) Y_l^m(\widehat{\mathbf{q}}). \tag{S14}
$$

This equation is evaluated numerically on an real-space grid,

$$c_{nlm}(\mathbf{r}) = i^l \sum_{\mathbf{q}} e^{i\mathbf{q}\mathbf{r}} n(\mathbf{q}) \tilde{\phi}_{nl}(q) Y_l^m(\widehat{\mathbf{q}}). \tag{S15}$$

Thus, the coefficients $c_{nlm}(\mathbf{r})$ can be calculated *simultaneously for all real-space gridpoints* via Fourier transform, and FFT achieves $\mathcal{N}_{\text{FFT}} \ln \mathcal{N}_{\text{FFT}}$ scaling with respect to the number of real-space grid points N_{FFT}.

To form descriptors for ML, one seeks rotationally invariant combinations of the coefficients c_{nlm}, as discussed by Bartok *et al.*[S4] The c_{n00} are already rotationally invariant, following from the fact that $Y_0^0 = 1/\sqrt{4\pi}$ is scalar. This gives us the two-body descriptors, $X_n^{(2)} = c_{n00}$. Further rotational invariants can be formed as follows[S2,S4]

$$p_{nn'l} = \sqrt{\frac{8\pi^2}{2l+1}} \sum_m c_{nlm} c_{n'lm}. \tag{S16}$$

For $l = 1$, these rotational invariants can be understood as all possible dot products between vectors $\mathbf{c}_n = \{c_{n1x}, c_{n1y}, c_{n1z}\}$. That the $\mathbf{c}_n$ are vectors follows from the vector property of $\mathbf{Y}_1 = \{Y_1^x, Y_1^y, Y_1^z\}$ (we use the real spherical harmonics, where this vector property is clearly manifest)

$$\begin{aligned} Y_1^x &= \sqrt{\frac{3}{4\pi}} \frac{x}{\sqrt{x^2+y^2+z^2}} \\ Y_1^y &= \sqrt{\frac{3}{4\pi}} \frac{y}{\sqrt{x^2+y^2+z^2}} \\ Y_1^z &= \sqrt{\frac{3}{4\pi}} \frac{z}{\sqrt{x^2+y^2+z^2}}. \end{aligned} \tag{S17}$$

To form three-body descriptors that have the same dimension as the $X_n^{(2)}$, we first form descriptors $X_{nn'l}^{(3)}$,

$$X_{nn'l}^{(3)} = \frac{\sigma^{(3)}}{R_{\text{cut}}} \frac{\sum_m c_{nlm} c_{n'lm}}{(\sum_m c_{nlm}^2)^{1/4} (\sum_m c_{n'lm}^2)^{1/4}}. \tag{S18}$$

Next, we limit the $X_{nn'l}^{(3)}$ to $l = 1$ and neglect off-diagonal elements $n \neq n'$, obtaining the

three-body descriptors $X_n^{(3)}$ [compare Eq. (4) in the main text]

$$\begin{aligned} X_n^{(3)} &= X_{nn'l}^{(3)} \delta_{nn'} \delta_{l1} \\ &= \frac{\sigma^{(3)}}{R_{\text{cut}}} \sqrt{c_{n1x}^2 + c_{n1y}^2 + c_{n1z}^2}. \end{aligned} \tag{S19}$$

We have verified that this last approximation works well for the application to diamond and liquid water. Generally, however, the validity of the approximation depends on the concrete training set as well as on the radial basis functions used.[S4] Here, we use four radial basis functions for both $l = 0$ and $l = 1$, thus we have in total $4 + 4 = 8$ density descriptors. Note that we use the weight factor $\sigma^{(3)}/R_{\text{cut}}$ in Eq. (S18), rather than $(8\pi^2/3)^{1/4}$ which would correspond to $\sqrt{p_{nn1}}$. The possibility of using different weight factors for the three-body descriptors was also discussed in detail by Jinnouchi et al.[S5] We have also tested additional three-body descriptors corresponding to higher angular quantum numbers [$l \geq 2$ in Eq. (S18)], but found no improvement. Again, the truncation at $l = 1$ used here is not necessarily optimal for other training sets. For instance, we speculate that higher angular quantum numbers might be required for transition metals. We have not attempted to implement four-body or higher-body density descriptors.

S1.1 Approaching GGA in the small cutoff limit

In Eqs. (3) and (5) in the main text, we have stated that in the limit of small cutoffs, the two-and three-body descriptors reduce to the local density and its gradient, respectively. A detailed proof of these limits is given in the following. For a small enough cutoff radius R_{cut}, the density inside the cutoff sphere varies little, assuming that the density is smooth enough. That means one can perform the gradient expansion of the density[S6] around $\mathbf{r}$

$$n(\mathbf{r} + \mathbf{r}') \approx n(\mathbf{r}) + r'_\alpha \nabla_\alpha n(\mathbf{r}) + \frac{1}{2} r'_\alpha r'_\beta \nabla_\alpha \nabla_\beta n(\mathbf{r}) + \frac{1}{6} r'_\alpha r'_\beta r'_\gamma \nabla_\alpha \nabla_\beta \nabla_\gamma n(\mathbf{r}) + ..., \tag{S20}$$

where α, β and γ are Cartesian indices and we use the Einstein sum convention. The same limit can also be obtained for fixed cutoff radius by considering artificial weakly varying densities ($\nabla n \to 0$, in the extreme case, one obtains the homogeneous electron gas). In other words, the dimensionless expansion parameter is $r'\nabla n$, and that can be small when either r' or ∇n is fixed and the other one goes to zero.

We insert the gradient expansion (S20) into the expression for the coefficients c_{nlm}, Eq. (S8), yielding

$$\begin{aligned} c_{nlm}(\mathbf{r}) \approx & \int_0^{R_{\text{cut}}} \mathrm{d}r'\ r'^2 \tilde{\phi}_{nl}(r') \int \mathrm{d}\Omega'\ Y_l^m(\widehat{\mathbf{r}'}) \\ & \times \left[n(\mathbf{r}) + r'_\alpha \nabla_\alpha n(\mathbf{r}) + \frac{1}{2} r'_\alpha r'_\beta \nabla_\alpha \nabla_\beta n(\mathbf{r}) + \frac{1}{6} r'_\alpha r'_\beta r'_\gamma \nabla_\alpha \nabla_\beta \nabla_\gamma n(\mathbf{r}) + ... \right]. \end{aligned} \tag{S21}$$

When evaluating the integral above, the first order term in the gradient expansion vanishes for $l = 0$. This is due to the anti-symmetry of the integrand. Likewise, the zeroth and second order terms vanish for $l = 1$. Thus, for the c_{n00} (two-body descriptors), Eq. (S21) simplifies to

$$c_{n00}(\mathbf{r}) \approx \sqrt{4\pi} \int_0^{R_{\text{cut}}} \mathrm{d}r'\ r'^2 \tilde{\phi}_{n0}(r') n(\mathbf{r}) + \frac{\sqrt{4\pi}}{6} \int_0^{R_{\text{cut}}} \mathrm{d}r'\ r'^4 \tilde{\phi}_{n0}(r') \nabla^2 n(\mathbf{r}) + ..., \tag{S22}$$

where we have used

$$\int \mathrm{d}\Omega'\ r'_\alpha r'_\beta = \frac{4\pi}{3} r'^2 \delta_{\alpha\beta}. \tag{S23}$$

Further, the radial integrals give numerical constants, the first being independent of R_{cut}, and the second being proportional to R_{cut}^2 (this can be seen by performing a variable transform $x = r'/R_{\text{cut}}$). The two-body descriptors in the limit of small cutoffs therefore have the expansion [compare Eq. (3) in the main text]

$$X_n^{(2)}(\mathbf{r}) \propto n(\mathbf{r}) + \text{const } \times R_{\text{cut}}^2 \nabla^2 n(\mathbf{r}) + ... \tag{S24}$$

Next, we use Eq. (S21) to approximate the $l = 1$ coefficients $c_{n1\alpha}$,

$$\begin{aligned} c_{n1\alpha}(\mathbf{r}) &\approx \int_0^{R_{\text{cut}}} \mathrm{d}r'\, r'^2 \tilde{\phi}_{n1}(r') \int \mathrm{d}\Omega'\, Y_1^{\alpha}(\widehat{\mathbf{r}'}) \left[r'_\beta \nabla_\beta n(\mathbf{r}) + \frac{1}{6} r'_\beta r'_\gamma r'_\delta \nabla_\beta \nabla_\gamma \nabla_\delta n(\mathbf{r}) + ... \right] \\ &= \sqrt{\frac{3}{4\pi}} \int_0^{R_{\text{cut}}} \mathrm{d}r'\, r' \tilde{\phi}_{n1}(r') \int d\Omega'\, r'_\alpha \left[r'_\beta \nabla_\beta n(\mathbf{r}) + \frac{1}{6} r'_\beta r'_\gamma r'_\delta \nabla_\beta \nabla_\gamma \nabla_\delta n(\mathbf{r}) + ... \right], \end{aligned} \tag{S25}$$

where in the second line we have plugged in Eq. (S17) for the Y_1^{α}. To simplify this expression, we use Eq. (S23) for leading order term and the identity

$$\int d\Omega' r'_\alpha r'_\beta r'_\gamma r'_\delta = \frac{4\pi}{15} r'^4 \left(\delta_{\alpha\beta}\delta_{\gamma\delta} + \delta_{\alpha\gamma}\delta_{\beta\delta} + \delta_{\alpha\delta}\delta_{\beta\gamma} \right) \tag{S26}$$

for next-to-leading order term, yielding

$$\begin{aligned} &c_{n1\alpha}(\mathbf{r}) \approx \\ &\sqrt{\frac{3}{4\pi}} \left\{ \left[\int_0^{R_{\text{cut}}} \mathrm{d}r'\, r'^3 \tilde{\phi}_{n1}(r') \right] \nabla_\alpha n(\mathbf{r}) + \frac{1}{10} \left[\int_0^{R_{\text{cut}}} \mathrm{d}r'\, r'^5 \tilde{\phi}_{n1}(r') \right] \nabla_\alpha \nabla_\beta \nabla_\beta n(\mathbf{r}) + ... \right\} \\ &\propto R_{\text{cut}} \nabla_\alpha n(\mathbf{r}) + \text{const} \times R_{\text{cut}}^3 \nabla_\alpha \nabla_\beta \nabla_\beta n(\mathbf{r}) + ... \end{aligned} \tag{S27}$$

Next, we form the scalar products $c_{n1\alpha} c_{n'1\alpha}$ needed for the three-body descriptors. The leading order term in the expansion of these scalar products is proportional to the scalar product $\nabla_\alpha n(\mathbf{r}) \nabla_\alpha n(\mathbf{r}) = |\boldsymbol{\nabla} n(\mathbf{r})|^2$, and the next-to-leading order term involves products of terms proportional to $\nabla_\alpha n(\mathbf{r}) \nabla_\alpha \nabla_\beta \nabla_\beta n(\mathbf{r}) = \boldsymbol{\nabla} n(\mathbf{r}) \cdot \boldsymbol{\nabla} \nabla^2 n(\mathbf{r})$, thus

$$c_{n1\alpha}(\mathbf{r}) c_{n'1\alpha}(\mathbf{r}) \propto R_{\text{cut}}^2 |\boldsymbol{\nabla} n(\mathbf{r})|^2 + \text{const} \times R_{\text{cut}}^4 \boldsymbol{\nabla} n(\mathbf{r}) \cdot \boldsymbol{\nabla} \nabla^2 n(\mathbf{r}) + ... \tag{S28}$$

Inserting this result in the definition (S18), we finally obtain

$$X_{nn'1}^{(3)}(\mathbf{r}) \propto |\boldsymbol{\nabla} n(\mathbf{r})| + \mathcal{O}(R_{\text{cut}}^2) \quad \text{for } R_{\text{cut}} \to 0. \tag{S29}$$

As the three-body descriptors are simply the diagonal elements ($n = n'$) of the $X_{nn'1}$, this

concludes the proof of the limit stated in Eq. (5) in the main text. Note that this limit extends also to the more general case of off-diagonal descriptors ($n \neq n'$).

Last, we briefly discuss how the ML-RPA descriptors could be generalized to collinear spin-DFT (we have not attempted to implement this, however). One first generalizes Eq. (S1) and expands the spin-densities n^{σ} to obtain expansion coefficients c^{σ}_{nlm}. The two-body descriptors c^{σ}_{n00} are straightforward extensions of the local spin-densities, compare Eq. (S24). Likewise, three-body descriptors are defined analogously to Eq. (S18). An important detail is that one has to consider also nondiagonal spin-terms ($\sigma \neq \sigma'$).

S2 Machine learning DFT via optimized effective potentials

In the following, we describe our ML scheme in more detail and discuss some challenges inherent to the use of optimized effective potentials (OEP). Further, we will derive analytic expression for the machine learned exchange-correlation potentials $v_{\mathrm{xc}}^{\mathrm{ML-RPA}}$ and show how they can be efficiently evaluated using FFTs.

We begin by briefly motivating our ML scheme via analogy to MLFFs. The starting point for MLFF schemes is the atomic density,

$$n_{\mathrm{atom}}(\mathbf{r}) = \sum_{i}^{\mathrm{atoms}} \delta(\mathbf{r} - \mathbf{R}_i). \tag{S30}$$

This atomic density is usually smoothed by replacing the delta function above by a Gaussian. The central assumption in MLFF schemes is that the total energy can by decomposed into a sum of atomic energies ε_i, which depend on two- and three-body descriptors (collected in a supervector $\mathbf{X}_{\mathrm{atom}}$),

$$E = \sum_{i}^{\mathrm{atoms}} \varepsilon_i[\mathbf{X}_{\mathrm{atom}}(\mathbf{R}_i)]. \tag{S31}$$

In DFT, the central quantity is the electronic density n. We do not apply smearing to n,

as the electronic density is already a smooth object. Analogously to Eq. (S31), one can formulate the assumption that the exchange-correlation energy can be written as an integral of energy densities depending on two- and three-body descriptors (supervector $\mathbf{X}$),

$$E_{\mathrm{xc}} = \int \mathrm{d}\mathbf{r}\; n(\mathbf{r})\varepsilon_{\mathrm{xc}}[\mathbf{X}(\mathbf{r})]. \tag{S32}$$

We further pull out a factor $\varepsilon_{\mathrm{x,HEG}}(\mathbf{r})$, such that the enhancement factor F_{xc} is approximated rather than $\varepsilon_{\mathrm{xc}}$. In other words, we use the LDA exchange as a baseline for ML-RPA, yielding the ansatz [Eq. (7) in the main text]

$$E_{\mathrm{xc}}^{\mathrm{ML-RPA}} = \int \mathrm{d}\mathbf{r}\; n(\mathbf{r})\varepsilon_{\mathrm{x,HEG}}[n(\mathbf{r})]F_{\mathrm{xc}}^{\mathrm{ML-RPA}}[\mathbf{X}(\mathbf{r})]. \tag{S33}$$

For the functional form of $F_{\mathrm{xc}}^{\mathrm{ML-RPA}}$, we use a Gaussian kernel [Eq. (8) in the main text]

$$F_{\mathrm{xc}}^{\mathrm{ML-RPA}}[\mathbf{X}(\mathbf{r})] = \sum_{i_B} w_{i_B} \exp\left\{-\frac{[\mathbf{X}(\mathbf{r}) - \mathbf{X}^{i_B}]^2}{2\sigma^2}\right\}, \tag{S34}$$

where the kernel width σ is an ML hyperparameter, the $\mathbf{X}^{i_B}$ are representative control points and the w_{i_B} the corresponding weights. Combing Eqs. (S33) and (S34), we obtain

$$E_{\mathrm{xc}}^{\mathrm{ML-RPA}} = \int \mathrm{d}\mathbf{r}\; n(\mathbf{r})\varepsilon_{\mathrm{x,HEG}}[n(\mathbf{r})] \sum_{i_B} w_{i_B} \exp\left\{-\frac{[\mathbf{X}(\mathbf{r}) - \mathbf{X}^{i_B}]^2}{2\sigma^2}\right\}. \tag{S35}$$

Evaluating the functional derivative, $v_{\mathrm{xc}}^{\mathrm{ML-RPA}} = \delta E_{\mathrm{xc}}^{\mathrm{ML-RPA}}/\delta n(\mathbf{r})$, yields a local term from the derivative of $n(\mathbf{r})\varepsilon_{\mathrm{x,HEG}}(\mathbf{r})$ and a nonlocal term which stems from the dependence of the descriptors $\mathbf{X}(\mathbf{r}')$ on the density $n(\mathbf{r})$

$$\begin{aligned} v_{\mathrm{xc}}^{\mathrm{ML-RPA}}(\mathbf{r}) &= \int \mathrm{d}\mathbf{r}' \frac{\delta}{\delta n(\mathbf{r})} \left\{n(\mathbf{r}')\varepsilon_{\mathrm{x,HEG}}[n(\mathbf{r}')]\right\} F_{\mathrm{xc}}^{\mathrm{ML-RPA}}(\mathbf{r}') \\ &+ \int \mathrm{d}\mathbf{r}' n(\mathbf{r}')\varepsilon_{\mathrm{x,HEG}}[n(\mathbf{r}')] \frac{\delta}{\delta n(\mathbf{r})} \left\{F_{\mathrm{xc}}^{\mathrm{ML-RPA}}[\mathbf{X}(\mathbf{r}')]\right\} \\ &= v_{\mathrm{xc,loc}}^{\mathrm{ML-RPA}}(\mathbf{r}) + v_{\mathrm{xc,nl}}^{\mathrm{ML-RPA}}. \end{aligned} \tag{S36}$$

The local term is easily evaluated using $\delta n(\mathbf{r}')/\delta n(\mathbf{r}) = \delta(\mathbf{r}-\mathbf{r}')$, yielding

$$
\begin{aligned}
v_{\text{xc,loc}}^{\text{ML-RPA}}(\mathbf{r}) &= \frac{4}{3}\varepsilon_{\text{x,HEG}}[n(\mathbf{r})]F_{\text{xc}}^{\text{ML-RPA}}[\mathbf{X}(\mathbf{r})] \\
&= \frac{4}{3}\varepsilon_{\text{x,HEG}}[n(\mathbf{r})]\sum_{i_B} w_{i_B}\exp\left\{-\frac{[\mathbf{X}(\mathbf{r})-\mathbf{X}^{i_B}]^2}{2\sigma^2}\right\}.
\end{aligned} \tag{S37}
$$

The nonlocal term is more complicated, but we will show in the following that FFT can be employed once again for its efficient evaluation. Using Eq. (S34) and applying the chain rule, we obtain

$$
\begin{aligned}
v_{\text{xc,nl}}^{\text{ML-RPA}}(\mathbf{r}) = \int & \mathrm{d}\mathbf{r}' n(\mathbf{r}')\varepsilon_{\text{x,HEG}}[n(\mathbf{r}')] \\
&\times \sum_{i_B} w_{i_B}\exp\left\{-\frac{[\mathbf{X}(\mathbf{r}')-\mathbf{X}^{i_B}]^2}{2\sigma^2}\right\}\sum_{i}^{N_{\text{des}}}\frac{-[X_i(\mathbf{r}')-\mathbf{X}^{i_B}]}{\sigma^2}\sum_{nml}\frac{\partial X_i(\mathbf{r}')}{\partial c_{nlm}(\mathbf{r}')}\frac{\delta c_{nlm}(\mathbf{r}')}{\delta n(\mathbf{r})}.
\end{aligned} \tag{S38}
$$

As the descriptors $X_i(\mathbf{r}')$ depend on the expansion coefficients $c_{nlm}(\mathbf{r}')$ in a simple algebraic fashion, the complicated nonlocality is thus due to the last term only. Inserting the expressions (S13) and (S10) yields

$$
\begin{aligned}
\frac{\delta c_{nlm}(\mathbf{r}')}{\delta n(\mathbf{r})} &= \frac{\delta}{\delta n(\mathbf{r})} i^l \int \frac{\mathrm{d}\mathbf{q}}{(2\pi)^3} e^{i\mathbf{q}\mathbf{r}'}\left[\int \mathrm{d}\mathbf{r}''\, e^{-i\mathbf{q}\mathbf{r}''} n(\mathbf{r}'')\right]\tilde{\phi}_{nl}(q)Y_l^m(\widehat{\mathbf{q}}) \\
&= i^l \int \frac{\mathrm{d}\mathbf{q}}{(2\pi)^3}\int \mathrm{d}\mathbf{r}''\, e^{i\mathbf{q}(\mathbf{r}'-\mathbf{r}'')}\delta(\mathbf{r}-\mathbf{r}'')\tilde{\phi}_{nl}(q)Y_l^m(\widehat{\mathbf{q}}) \\
&= i^l \int \frac{\mathrm{d}\mathbf{q}}{(2\pi)^3} e^{i\mathbf{q}(\mathbf{r}-\mathbf{r}')}\tilde{\phi}_{nl}(q)Y_l^m(-\widehat{\mathbf{q}}),
\end{aligned} \tag{S39}
$$

where in the last line we have substituted $\mathbf{q} \mapsto -\mathbf{q}$. Next, we define the intermediate quantities η_{nlm}, which we evaluate numerically via FFT,

$$
\begin{aligned}
\eta_{nlm}(\mathbf{q}) = \frac{1}{N_{\text{FFT}}} & \sum_{\mathbf{r}'} e^{-i\mathbf{q}\mathbf{r}'} n(\mathbf{r}')\varepsilon_{\text{x,HEG}}[n(\mathbf{r}')] \\
&\times \sum_{i_B} w_{i_B}\exp\left\{\frac{-[\mathbf{X}(\mathbf{r}')-\mathbf{X}^{i_B}]^2}{2\sigma^2}\right\}\sum_{i}^{N_{\text{des}}}\frac{-[X_i(\mathbf{r}')-\mathbf{X}^{i_B}]}{\sigma^2}\frac{\partial X_i(\mathbf{r}')}{\partial c_{nlm}(\mathbf{r}')}.
\end{aligned} \tag{S40}
$$

With the help of the η_{nlm}, we can rewrite Eq. (S38) in the compact form

$$v_{\text{xc,nl}}^{\text{ML-RPA}}(\mathbf{r}) = i^l \sum_{\mathbf{q}} e^{i\mathbf{q}\mathbf{r}} \sum_{nlm} \eta_{nlm}(\mathbf{q}) \tilde{\phi}_{nl}(q) Y_l^m(-\widehat{\mathbf{q}}), \tag{S41}$$

which can be directly evaluated via FFT as well. In summary, Eqs. (S40) and (S41) allow us to evaluate the ML-RPA exchange-correlation potential on all real-space grid points $\mathbf{r}$ using a small number of FFTs. Thus, we have *avoided the evaluation of double integrals* by applying FFT throughout. Therefore, the overall computational cost of evaluating $v_{\text{xc}}^{\text{ML-RPA}}$ scales only as $\mathcal{O}(N_{\text{FFT}} \ln N_{\text{FFT}})$ rather than (N_{FFT}^2) with respect to the number of real-space grid points N_{FFT}.

A potential pitfall in using the OEP method for ML applications is the fact that $v_{\text{xc}}^{\text{RPA}}(\mathbf{r})$ is in practice determined only up to a constant shift. Inspired by the work of Nagai et al.,[S7] we circumvent this problem by defining auxiliary exchange-correlation potentials $\tilde{v}_{\text{xc}}^{\text{RPA}}$,

$$\tilde{v}_{\text{xc}}^{\text{RPA}}(\mathbf{r}) = v_{\text{xc}}^{\text{RPA}}(\mathbf{r}) + \frac{E_{\text{xc}}^{\text{RPA}} - \int \mathrm{d}\mathbf{r}' n(\mathbf{r}') v_{\text{xc}}^{\text{RPA}}(\mathbf{r}')}{\int \mathrm{d}\mathbf{r}' n(\mathbf{r}')}. \tag{S42}$$

Thus, the $\tilde{v}_{\text{xc}}^{\text{RPA}}$ are shifted with respect to the $v_{\text{xc}}^{\text{RPA}}$ such that they integrate to $E_{\text{xc}}^{\text{RPA}}$,

$$\int \mathrm{d}\mathbf{r}\; n(\mathbf{r}) \tilde{v}_{\text{xc}}^{\text{RPA}}(\mathbf{r}) \stackrel{!}{=} E_{\text{xc}}^{\text{RPA}}. \tag{S43}$$

In fitting, we equate the auxiliary potentials with their ML-RPA analogs, $\tilde{v}_{\text{xc}}^{\text{ML-RPA}}$,

$$\tilde{v}_{\text{xc}}^{\text{ML-RPA}}(\mathbf{r}) = v_{\text{xc}}^{\text{ML-RPA}}(\mathbf{r}) + \frac{E_{\text{xc}}^{\text{ML-RPA}} - \int \mathrm{d}\mathbf{r}'\; n(\mathbf{r}') v_{\text{xc}}^{\text{ML-RPA}}(\mathbf{r}')}{\int \mathrm{d}\mathbf{r}'\; n(\mathbf{r}')}. \tag{S44}$$

Thus, any information regarding absolute values of the OEP potentials is circumvented. Similar shifted exchange-correlation potentials occur also in the ML scheme of Tozer *et al.*,[S8] the Becke-Johnson method[S9] and the Levy-Zahariev formulation of DFT.[S10] Here, however, the auxiliary potentials are used only as intermediate quantities for fitting, and once an

ML-RPA functional has been learned, standard exchange-correlation potentials $v_{\text{xc}}^{\text{ML-RPA}}$ are extracted for applications.

To find the weights w_{i_B}, we fit to exchange-correlation energies and shifted exchange-correlation potentials at selected points $\mathbf{r}_k$ for all structures α contained in the training set. We demand that ML-RPA reproduces the reference data in a least square sense and apply appropriate weights, yielding the loss function

$$\begin{aligned}
\mathcal{L}_2 &= \frac{1}{N_{\text{struct}}} \sum_{\alpha}^{N_{\text{struct}}} c_E \mathcal{L}_{2,E}^{\alpha} + (1 - c_E)\mathcal{L}_{2,v}^{\alpha} \\
\mathcal{L}_{2,E}^{\alpha} &= \frac{1}{2}\frac{1}{N_e^{\alpha}} \left(E_{\text{xc}}^{\text{ML-RPA}} - E_{\text{xc}}^{\text{RPA}}\right)^2 \\
\mathcal{L}_{2,v}^{\alpha} &= \frac{1}{2}\frac{1}{N_e^{\alpha}}\frac{\Omega^{\alpha}}{N_{\text{spars}}} \sum_{k}^{N_{\text{spars}}} n(\mathbf{r}_k) \left[\tilde{v}_{\text{xc}}^{\text{ML-RPA}}(\mathbf{r}_k) - \tilde{v}_{\text{xc}}^{\text{RPA}}(\mathbf{r}_k)\right]^2 .
\end{aligned} \tag{S45}$$

Here we have introduced a dimensionless weight factor c_E, which allows us to balance exchange-correlation energies and potentials. Further, we normalize the loss with respect to system size via dividing by the number of electrons N_e^{α}. Likewise, a factor $\Omega^{\alpha}/N_{\text{spars}}^{\alpha}$ is included for the exchange-correlation potentials, where Ω^{α} is the volume of structure α. From Eq. (S42) it is clear that the shifted exchange-correlation potentials depend linearly on the w_{i_B} just as the unshifted ones do. Thus, we can solve a system of linear equations that is obtained via minimization of the loss function (S45) with respect to the w_{i_B},

$$\partial \mathcal{L}_2 / \partial w_{i_B} \stackrel{!}{=} 0 \rightarrow \sum_{i_B} \phi_{j,i_B}^{\alpha} w_{i_B} = y_j^{\alpha}. \tag{S46}$$

Following Verdi et al.,[S11] we regularize the solution of this linear problem via pseudo inverse of the design matrix ϕ, smoothly cutting off smaller singular values σ_i,

$$\sigma_i^{-1} \mapsto \frac{\sigma_i}{\sigma_i^2 + (t_{\text{SVD}}\sigma_{\max})^2}, \tag{S47}$$

where we multiply the Tikhonov parameter t_{SVD} by the largest singular value $\sigma_{\max}$. Thus,

t_{SVD} is dimensionless and we can more easily compare numerical values of t_{SVD} for different databases.

S2.1 Data sparsification

To reduce computational cost, the exchange-correlation potential is fitted not on the entire real-space grid but rather at selected representative points $\mathbf{r}_k$. These points are represented as red crosses in Figure 1 in the main text. For each individual structure α, we choose N_{spars} points via k-means sparsification. The k-means algorithm uses a metric that quantifies the similarity between density descriptors at points $\mathbf{r}$ and $\mathbf{r}'$. It is convenient to use the metric $d[\mathbf{X}(\mathbf{r}),\mathbf{X}(\mathbf{r}')]$ that is induced by the Gaussian kernel, $k[\mathbf{X}(\mathbf{r}),\mathbf{X}(\mathbf{r}')]$,

$$\begin{aligned} d[\mathbf{X}(\mathbf{r}),\mathbf{X}(\mathbf{r}')] &= k[\mathbf{X}(\mathbf{r}),\mathbf{X}(\mathbf{r})] + k[\mathbf{X}(\mathbf{r}'),\mathbf{X}(\mathbf{r}')] - 2k[\mathbf{X}(\mathbf{r}),\mathbf{X}(\mathbf{r}')] \\ &= 2 - 2\exp\left\{-\frac{[\mathbf{X}(\mathbf{r}) - \mathbf{X}(\mathbf{r}')]^2}{2\sigma^2}\right\}. \end{aligned} \tag{S48}$$

The k-means centers are initialized via farthest point sampling similar to ref S12. In further iterations, the centers are updated as averages over all points belonging to their respective clusters as in the standard k-means algorithm. Those points are assigned to the clusters based again on the kernel induced metric.

Next, we combine the selected points from all structures and apply the sparsification again to choose the kernel control points $\mathbf{X}^{i_B}$, compare blue squares in Figure 1 in the main text. An interesting technical detail is that the $\mathbf{r}_k$ are chosen as *actual real-space points* closest to k-means centers, where $v_{\mathrm{xc}}^{\mathrm{RPA}}(\mathbf{r})$ is available. For the selection of the $\mathbf{X}^{i_B}$, however, we find it beneficial to use *the k-means centers themselves.* That is, the chosen kernel control points correspond not to descriptors at actual real-space grid points, but rather optimized artificial ones. Integrals are evaluated on the entire real-space grid throughout.

In the following, we demonstrate numerically the efficiency of our sparsification scheme. First, we split the ML-RPA training set randomly (50:50) into a reduced training set and

a validation set. Keeping one sparsification layer fixed and varying the number of k-means clusters in the other, we monitor the loss

$$\begin{aligned}\mathcal{L}_1{}' &= \frac{1}{N_{\text{struct}}}\sum_{\alpha}^{N_{\text{struct}}} c_E \mathcal{L}_{1,E}^{\alpha} + (1-c_E)\mathcal{L}_{1,v}^{\alpha}{}' \\ \mathcal{L}_{1,E}^{\alpha} &= \frac{1}{N_e^{\alpha}}|E_{\text{xc}}^{\text{ML-RPA}} - E_{\text{xc}}^{\text{RPA}}| \\ \mathcal{L}_{1,v}^{\alpha}{}' &= \frac{1}{N_e^{\alpha}}\int \mathrm{d}\mathbf{r}\; n(\mathbf{r})|\tilde{v}_{\text{xc}}^{\text{ML-RPA}}(\mathbf{r}) - \tilde{v}_{\text{xc}}^{\text{RPA}}(\mathbf{r})|\end{aligned} \tag{S49}$$

for structures in the reduced training and validation sets. Note that the loss $\mathcal{L}_{1,v}^{\alpha}{}'$ includes the exchange-correlation potential *at all real-space grid points*, thus some amount of interpolation is required to minimize $\mathcal{L}_{1,v}^{\alpha}{}'$ even for structures α on which ML-RPA has been trained on. This means that $\mathcal{L}_{1,v}{}'$ is less prone to overfitting and statistical error. Likewise, atomic forces in MLFFs are known to be less prone to overfitting and statistical errors than energies. Figure S1 shows that in the first layer, we can downsample the number of real-space grid points per training structure (N_{spars}) from $\mathcal{O}(10^5) - \mathcal{O}(10^6)$ to a mere 100 without loosing significant fit accuracy. The second k-means layer inputs the combined $N_{\text{struct}} \times N_{\text{spars}}$ points from the first layer. Figure S2 shows that the number of kernel control points can be reduced by an additional factor of 4 without loss of accuracy.

Finally, we briefly comment on the importance of sparsification for ML-RPA performance. The evaluation of the ML-RPA descriptors is exceedingly fast, see discussion after Eq. (S15). This leaves our current implementation with two main bottlenecks: (i) the FFTs required for the evaluation of $v_{\text{xc}}^{\text{ML-RPA}}$ [Eq. (S41)], and (ii) the repeated evaluation of the Gaussian kernel that is also required for $E_{\text{xc}}^{\text{ML-RPA}}$ [Eq. (S35)]. This second bottleneck scales linearly with the number of kernel control points, and thus benefits directly from sparsification. We feel that the current level of sparsification is already fairly satisfactory, since it brings the down the computational cost roughly to that of bottleneck (i). Nevertheless, bottleneck (ii) could be eliminated either by replacing the Gaussian kernel with a neural network, or via kernel interpolation techniques (see for example ref S13).

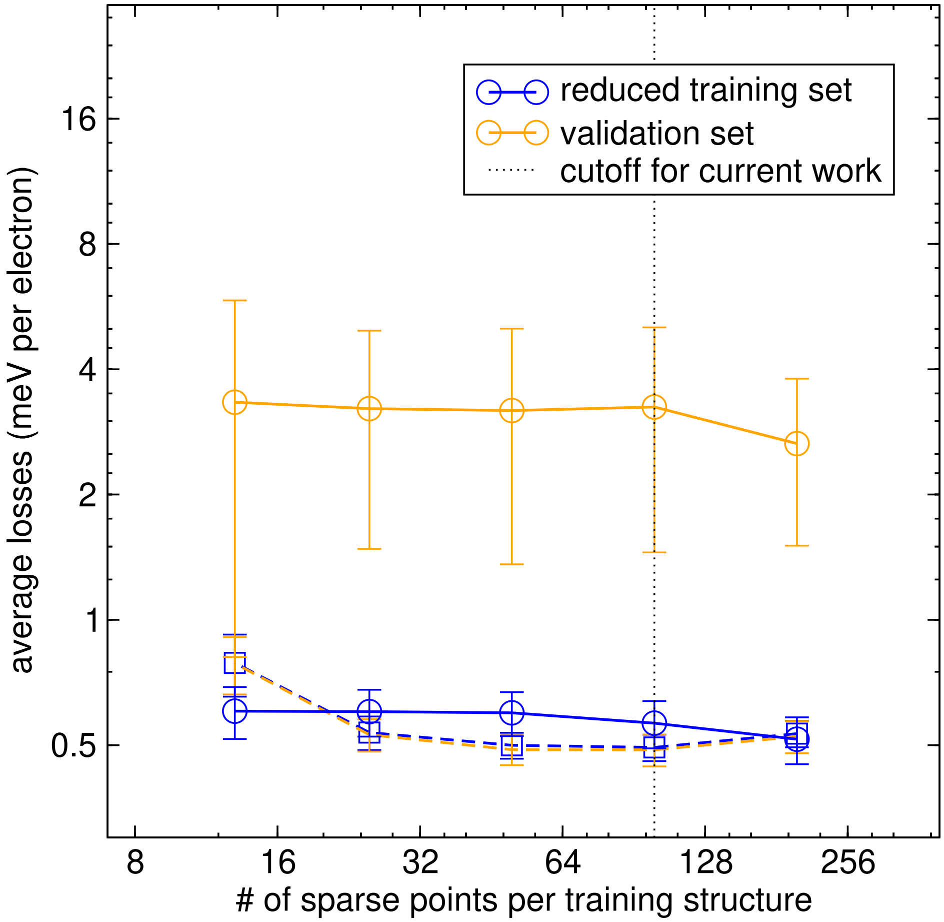

Figure S1: Convergence of the ML-RPA fit with respect to N_{spars}, that is the number of sparse points kept by the first sparsification layer (second layer fixed). Note the log-log scale. Solid lines indicate losses for exchange-correlation energies [$c_E \mathcal{L}_E$ in Eq. (S49)], and dashed lines indicate losses for exchange-correlation potentials [$(1 - c_E)\mathcal{L}'_v$ in Eq. (S49)]. The losses are averaged losses over 10 random splittings (50:50).

It is interesting to note that the need for functional derivatives [and thus bottleneck (i)] can in principle be sidestepped when one is working completely within the Levy-Zahariev formulation of DFT.[S10] In fact, we have briefly attempted such an ML scheme, where one learns directly the shifted exchange-correlation potentials $\tilde{v}_{\text{xc}}(\mathbf{r})$ [Eq. (S42)] instead of the enhancement factor $F_{\text{xc}}(\mathbf{r})$ [compare Eq. (S33)]. However, this scheme performs worse than the ansatz (S36) that manifestly enforces the relation $v_{\text{xc}}^{\text{ML-RPA}}(\mathbf{r}) = \delta E_{\text{xc}}^{\text{ML-RPA}} / \delta n(\mathbf{r})$ (not shown).

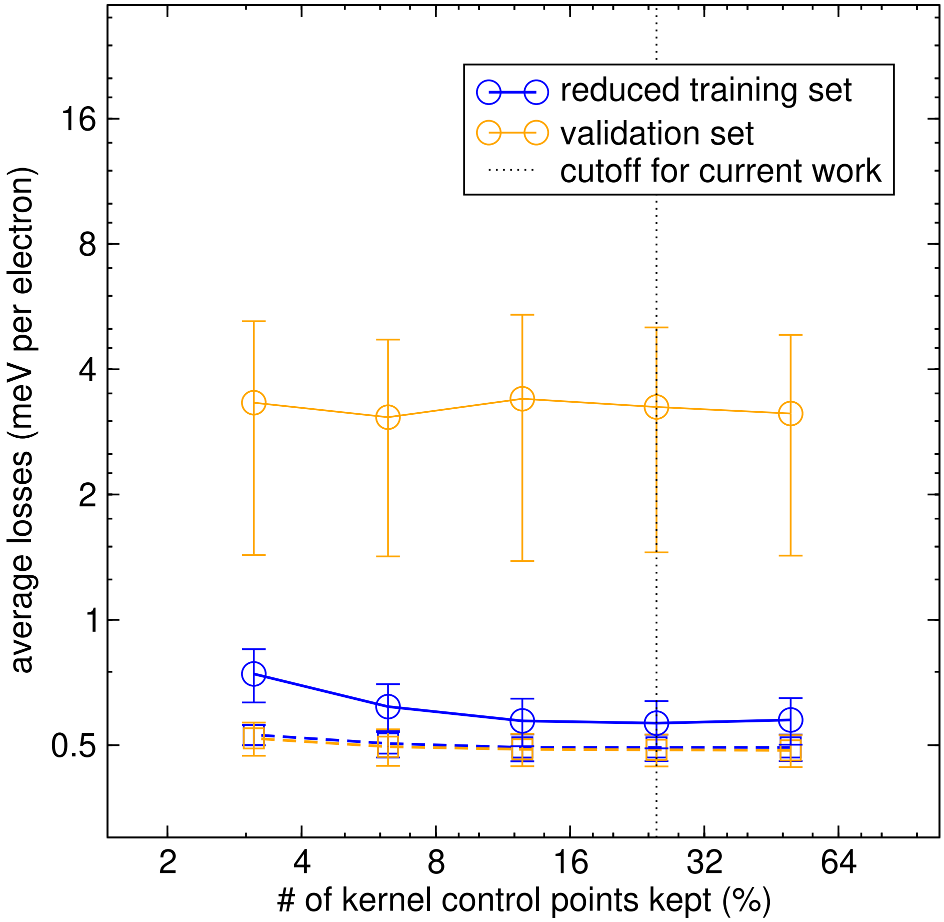

Figure S2: Like Figure S1, but convergence with respect to the number of kernel control points kept by the second sparsification layer (first layer fixed).

S2.2 ML-RPA hyperparameters

The ML-RPA hyperparameters are given in Table S1. The hyperparameter values were optimized by minimizing validation set losses and monitoring the stability of electronic self-consistency. Training set losses for the standard ML-RPA hyperparameters are detailed in Table S2 with respect to the different training set subgroups. In the following, we briefly discuss some important hyperparameter choices. We focus on the training set losses for the exchange-correlation potentials $[(1 - c_E)\mathcal{L}'_{1,v}]$ that generalize well to unseen structures (see Figures S1 and S2).

First, we discuss the choice of the cutoff radius R_{cut}, which determines the nonlocality range for ML-RPA. That is, at a given point $\mathbf{r}$, the descriptors "see" the density $n(\mathbf{r} + \mathbf{r}')$

Table S1: Table of ML-RPA hyperparameters.

parameter	description	value
R_{cut}	cutoff radius	1.5 Å
N_{rad}	number of radial basis functions	4
N_{des}	number of density descriptors	4+4=8
σ	width of Gaussian kernel	3.0 eÅ^{-3}
$\sigma^{(3)}$	weight for three-body descriptors	1.0 Å
c_E	fit weight for xc-energies	0.999
t_{SVD}	Tikhonov regularization	1.0×10^{-9}

Table S2: Average training set losses calculated via Eq. (S49) (in meV per electron) specified for different subgroups of the ML-RPA training set, with hyperparameters as detailed in Tab. S1. To compensate for the small amount of surface data, surfaces are included twice in the training set, giving them higher fit weight. The H_2O monomer in the experimental geometry is listed as a G2 molecule.

	$c_E\mathcal{L}_{1,E}$	$(1-c_E)\mathcal{L}_{1,v}{}'$
41 G2-molecules	0.73	0.30
40 bulk diamond structures	0.59	0.48
16 diamond surfaces (x2)	0.93	1.09
76 water structures	0.71	0.27
189 structures in total	0.73	0.46

up to distances $r' \leq R_{\text{cut}}$ [see Eq. (S2)]. In the main text, we point out that our choice of $R_{\text{cut}} = 1.5$ Å is still fairly short-ranged. However, when R_{cut} is increased to 2.0 Å and further to 3.0 Å (keeping the other hyperparameters fixed), the exchange-correlation potential loss increases from 0.46 meV per electron to 0.60 meV per electron and 0.62 meV per electron, respectively. We have confirmed that larger cutoffs also lead to increasing ML-RPA force errors for liquid water (not shown). Arguably, one should allow for the number of radial basis functions N_{rad} to increase alongside R_{cut}. Even then, we find that the setup listed in Table S2 is optimal.

Next, we discuss the hyperparameter c_E that weighs losses for exchange-correlation energies versus losses for exchange-correlation potentials [see Eqs. (S45) and (S49)]. One might be tempted to assume that 1/2 would be the natural parameter value, however, we find that

values of c_E closer to 1 are optimal (here we use $c_E = 0.999$). This is due to the fact that through the modulus $|\tilde{v}_{\text{xc}}^{\text{ML-RPA}}(\mathbf{r}) - \tilde{v}_{\text{xc}}^{\text{RPA}}(\mathbf{r})|$, the respective exchange-correlation potentials have to agree *at each real-space grid point* $\mathbf{r}$. In contrast, the exchange-correlation energy is an integrated quantity that can benefit from error cancellation. In this sense, the loss functions adapted for the exchange-correlation potentials are stricter than the corresponding losses for the exchange-correlation energies. We find that it is beneficial to increase the Tikhonov regularization parameter t_{SVD} alongside c_E. This makes sense if we picture the exchange-correlation potentials as regularizer for the exchange-correlation energies. For example, we increase c_E from 0.999 to 0.9999, and simultaneously increase t_{SVD} from 10^{-9} to 10^{-8}. Going in the other direction, we decrease c_E to 0.99 and simultaneously decrease t_{SVD} to 10^{-10}. This changes the exchange-correlation potential loss from 0.48 meV per electron to 0.09 meV per electron and 3.97 meV per electron, respectively. However, the optimization of c_E in terms of these losses is not so clear since c_E enters the loss function explicitly. The trend would reverse if the losses $\mathcal{L}_{1,v}{}'$ were not multiplied by $(1 - c_E)$. That is, if we measure the unweighed losses $\mathcal{L}_{1,v}{}'$ (and use c_E as a weight factor in fitting only), the exchange-correlation potentials are more accurate for smaller c_E, which is the behavior that one would also initially expect. To avoid this ambiguity, we have optimized c_E by minimizing only the unweighed validation set losses for exchange-correlation energies $\mathcal{L}_{1,E}$.

S3 Diamond surfaces

Table S3 specifies the 28 diamond surfaces used to benchmark different DFT functionals (see Table 2 in the main text). These surfaces have been described in detail in past studies, we refer to the original references for more complete descriptions of the surface geometries.[S16,S17,S19–S21] In the following, we briefly comment on the interesting case of oxygenated (111) surfaces, where several (meta-)stable configurations exist that are close in energy. The least stable surface is the (111)-3db symmetric (1×1) oxygenated surface, while the most

Table S3: List of the 28 diamond surfaces used for our surface energy benchmark (see Table 2 in the main text). The first column defines the surface symmetry and stoichiometry (corresponding to the chemisorption of 1 ML hydrogen for hydrogenated surfaces, and 1 ML oxygen for oxygenated surfaces). The second column describes the geometry of the surface termination and quotes literature references, where the surfaces are characterized. Further columns show surface formation energies [see Eq. (12) in the main text] that are given in eV per surface atom. RPA is the ground truth for ML-RPA, and basis set extrapolated RPA formation energies are obtained using Eq. (S50). Formation energies calculated with the PBE functional are also listed for comparison. All (100) and (111)-1db surfaces are included in the ML-RPA training set, all (110) and (111)-3db surfaces are out-of training. Underlined values correspond to the most stable configurations for a given a surface termination and orientation.

		PBE	extrap.	RPA	ML-RPA
**(100)***					
1×1	bulk terminated, as cut[S14]	3.49	3.70	3.66	3.57
1×1	bulk terminated, relaxed[S14]	3.36	3.62	3.59	3.51
1×1:H	bulk terminated, on-top[S14]	0.90	1.21	1.21	1.16
1×1:O	bulk terminated, ketone[S15]	2.29	2.38	2.38	2.39
1×1:O	bulk terminated, ether[S15]	1.97	2.03	1.99	1.91
2×1	dimer[S15]	1.89	2.08	2.08	2.03
2×1:2H	dimer, on-top[S15]	0.01	0.20	0.20	0.11
2×1:2O	dimer, bridge[S16]	3.45	3.50	3.48	3.40
**(110)**					
1×1	bulk terminated, as cut[S17]	1.90	2.11	2.09	1.95
1×1	bulk terminated, relaxed[S17]	1.45	1.69	1.70	1.59
1×1:H	bulk terminated, on-top[S17]	-0.23	-0.12	-0.11	-0.12
1×1:O	bulk terminated, on-top†	3.38	3.61	3.60	3.58

*included in the ML-RPA training set

†similar to the hydrogenated (110) surface, the calculated C-O bond length is 1.36 Å.

stable surface is the 2×1 reconstructed (111)-3db oxygenated chain surface, and the three oxygenated (111)-1db surfaces are in between. The (111)-3db oxygenated chain surface can be interpreted as clean (111)-1db surface adsorbing a monolayer of CO molecules, compare Figure 6 in the main text. This is significant insofar as CO molecules have been reported to be the main desorption product in temperate-programmed desorption experiments on oxygenated (111) surfaces.[S19] The C-O bond length of the oxygenated (111)-3db chain surface is calculated to be 1.20 Å (we use PBE geometries throughout). This clearly indicates strong

Table S3, continued.

		PBE	extrap.	RPA	ML-RPA
**(111)-1db***					
1×1	bulk terminated, as cut[S18]	2.46	2.71	2.69	2.52
1×1	bulk terminated, relaxed[S18]	1.94	2.30	2.29	2.12
1×1:H	bulk terminated, on-top[S18]	-0.34	-0.19	-0.19	-0.24
1×1:O	bulk terminated, on-top[S19]	2.84	3.19	3.19	2.99
2×1:2O	bulk terminated (distorted), peroxide[S19]	2.99	3.02	2.99	2.94
2×1	Pandey chain[S18]	1.18	1.43	1.41	1.34
2×1:2H	Pandey chain, on-top[S18]	0.31	0.49	0.47	0.40
2×1:2O	Pandey chain, ketone[S19]	2.94	3.08	3.09	2.99
**(111)-3db**					
1×1	bulk terminated, as cut[S20]	4.27	4.41	4.37	4.37
1×1	bulk terminated, relaxed[S20]	4.26	4.41	4.37	4.37
1×1:H	bulk terminated, on-top[S20]	3.01	3.38	3.36	3.43
1×1:O	bulk terminated, ketone[S16]	3.50	3.92	3.90	3.87
2×1	Seiwatz chain[S20]	2.44	2.71	2.70	2.56
2×1:2H	Seiwatz chain, on-top[S20]	0.00	0.17	0.17	0.11
2×1:2O	Seiwatz chain, ketone[S16]	2.67	2.91	2.90	2.79

*included in the ML-RPA training set

double bonds, in comparison, the C-O bond lengths for the (111)-1db oxygenated surfaces are 1.20 Å (ketone) and 1.31 Å (on-top), as well as $\{1.43\ \text{Å}, 1.44\ \text{Å}\}$ (peroxide). Out of the three oxygenated (111)-1db surfaces, the RPA and ML-RPA predict the peroxide structure to be the most stable one, whereas PBE favors the on-top configuration. Otherwise, PBE, ML-RPA and the RPA predict identical energy orderings throughout, see underlined values in Table S3.

Finally, correlated methods such as the RPA are known to converge slowly with respect to the basis set size.[S22,S23] To confirm the technical converge of our RPA calculations with respect to basis set, we extrapolate the RPA correlation energies with respect to the energy cutoff E^{χ}_{max} using the formula[S24,S25]

$$E_{\text{c}}^{\text{RPA}}(E^{\chi}_{\text{max}}) = E_{\text{c}}^{\text{RPA}}(E^{\chi}_{\text{max}} = \infty) + \frac{\text{const}}{{E^{\chi}_{\text{max}}}^{3/2}}. \tag{S50}$$

Table S3 shows that the extrapolated formation energies are slightly larger than our "RPA" values, which form the ground truth for ML-RPA. The agreement between RPA and extrapolated RPA formation energies is 40 meV or better throughout.

The basis set incompleteness error also causes a slight underbinding of bulk diamond. The extrapolated value for the equilibrium lattice constant is 3.572 Å, whereas RPA without basis set correction predicts 3.581 Å.

S4 Machine learning force fields for liquid water

RPA-MLFF is trained directly on total energies and forces from RPA calculations. Since RPA force calculations are very expensive, we use a slightly cheaper RPA setup here. Specifically, an energy cutoff of 500 eV is used for the plane-wave basis sets expanding both the orbitals and the response function (ML-RPA ground truth calculations use 600 eV and 400 eV, respectively). Our tests, however, indicate that this difference has only minor effects on the predicted liquid water RDFs. For instance, the forces typically differ by 10 meV Å^{-1} (root mean square error). Moreover, the equilibrium volumes of I_c(a) cubic ice are very similar (32.64 Å^3 per H_2O for the ML-RPA ground truth vs 32.75 Å^3 per H_2O for the RPA-MLFF ground truth), and further in close agreement to the basis set extrapolated value of 32.77 Å^3 per H_2O (reported by Macher et al.,[S26] who also used plane wave basis sets). In contrast, the equilibrium ice volume reported by Del Ben et al.[S27] who used a similar computational setup as Yao and Kanai[S28] is somewhat smaller (32.37 Å^3 per H_2O for hexagonal ice I_h that is very similar to cubic ice[S26]). In particular, we believe that their the use of a triple-zeta local basis set might be insufficient. To this point, a detailed study by Eshuis and Furche[S29] showed that quadrupole-zeta quality basis sets are typically required to converge RPA calculations. Furthermore, we could also obtain smaller ice equilibrium volumes by reducing the plane-wave cutoff for the response function (though, of course, this is not a rigorous proof, since plane-wave and local basis sets are not directly comparable).

The RPA-MLFF training data set consists 107 water structures containing 32 molecules or less. We use a compact hyperparameter setup that has shown to enable an efficient and accurate MLFF training for liquid water.[S30] In particular, the radial and angular descriptors are separated as described in ref S5, and the angular descriptors are truncated at angular momentum number $l = 2$. Further, the radial descriptors use a cutoff of 6.0 Å and 8 radial basis functions, whereas the angular descriptors use a smaller cutoff of 4.0 Å and only 6 radial basis functions. For a general description of the MLFF scheme see also refs S31 and S2. The RPA-MLFF training set errors are given in Table S4.

Table S4: Training set errors (root means square error, RMSE) for the MLFFs trained for liquid water. Energy RMSEs are given in meV per atom, force RMSEs are given in meV Å^{-1}, and stress RMSEs are given in kbar.

	structures	energy	force	stress
RPA-MLFF	107	0.6	27.0	—
ML-RPA-MLFF	389	0.4	29.9	0.26
PBE+TS-MLFF	490	0.3	31.7	0.21
RPBE+D3(BJ)-MLFF	521	0.3	30.5	0.21

The MLFF used to speed up ML-RPA uses the same descriptors as RPA-MLFF, but is trained on-the-fly[S2,S31] at fixed volume using a temperature ramp from 270 K to 370 K and a supercell containing 64 water molecules. In this way, a training set of around 400 structures is created. Thus, the combination of ML-RPA and MLFFs allows for significantly more MLFF ground truth calculations, since ML-RPA is orders of magnitude cheaper than the RPA. Moreover, the stresses predicted by ML-RPA can be seamlessly included in the MLFF training, whereas this would not be as easily possible for RPA-MLFF (RPA stress tensors can be included via finite differences,[S32] but this is very expensive). The MLFFs for PBE+TS and RPBE+D3(BJ) are also trained on-the-fly, fit accuracy being similar to ML-RPA (see Table S4).

The production run which produced the RDFs shown in Figure 7 in the main text used 200000 MD steps with a time step of 1.5 fs. To increase sampling efficiency, the mass of the

hydrogen atom was increased by a factor 8 (this does not affect static properties such as the RDF). For PBE+TS, which diffuses very weakly at 300 K, we further increased the supercell size to 512 water molecules, and increased the length of the MD run by a factor 3. Next, we define an independent test set of 10 liquid water snapshots sampled from the RPA-MLFF production run (64 water molecules). A low test set energy root means square error (RMSE) of 1.6 meV per atom demonstrates (i) that the ambient conditions used are well covered by the RPA-MLFF training set, (ii) that RPA-MLFF is able to extrapolate to the slightly larger simulation cell (64 water molecules). We also use this test set to evaluate the on-the-fly MLFFs, and the test set energy RMSEs are excellent (0.4 meV per atom for ML-RPA, 0.6 meV per atom for PBE+TS, and 0.5 meV per atom for RPBE+D3(BJ), respectively).

S5 Water hexamer benchmark

Figures S3 and S4 detail the water hexamer benchmark for various semilocal and vdW functionals, respectively. The vdW corrected PBE+TS and RPBE+D3 functionals well improve over their respective GGA base functionals, whereas SCAN+rVV10 performs slightly worse than the pristine SCAN functional.

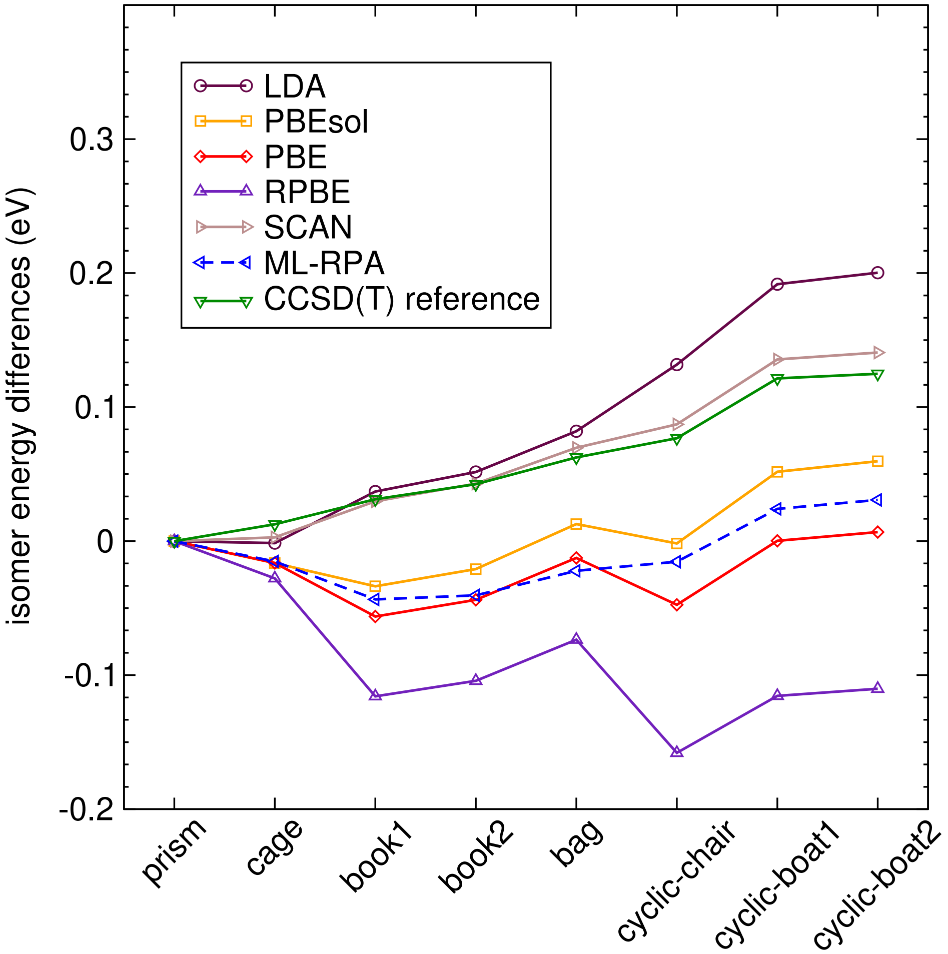

Figure S3: Binding energies differences of eight hexamers as in Figure 8 in the main text. ML-RPA results are compared to various semilocal density functionals. Lines drawn are only guides to the eye.

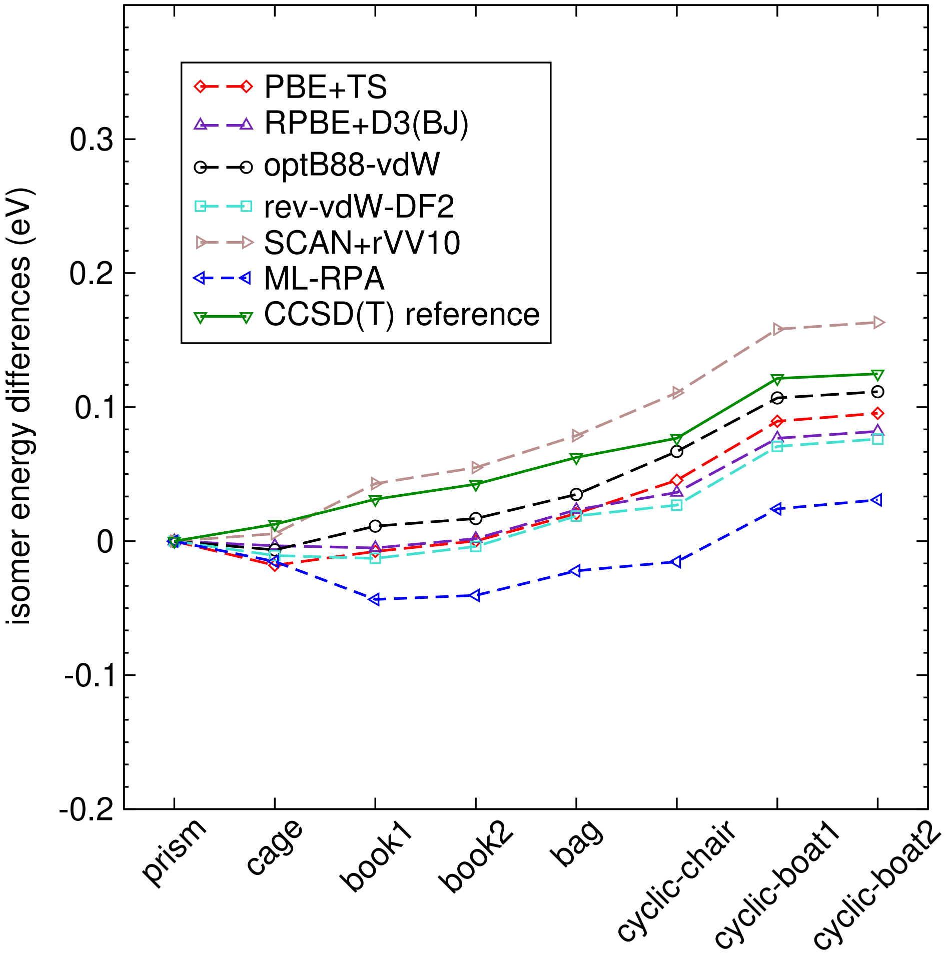

Figure S4: Like Figure S3, but for various vdW functionals.

References

(S1) Behler, J.; Parrinello, M. Generalized Neural-Network Representation of High-Dimensional Potential-Energy Surfaces. *Physical Review Letters* **2007**, *98*, 146401.

(S2) Jinnouchi, R.; Karsai, F.; Kresse, G. On-the-fly machine learning force field generation: Application to melting points. *Physical Review B* **2019**, *100*, 014105.

(S3) Arfken, G. B.; Weber, H. J. Mathematical methods for physicists. 1999.

(S4) Bartók, A. P.; Kondor, R.; Csányi, G. On representing chemical environments. *Physical Review B* **2013**, *87*, 184115.

(S5) Jinnouchi, R.; Karsai, F.; Verdi, C.; Asahi, R.; Kresse, G. Descriptors representing two- and three-body atomic distributions and their effects on the accuracy of machine-learned inter-atomic potentials. *The Journal of Chemical Physics* **2020**, *152*, 234102.

(S6) Kohn, W.; Sham, L. J. Self-Consistent Equations Including Exchange and Correlation Effects. *Phys. Rev.* **1965**, *140*, A1133–A1138.

(S7) Nagai, R.; Akashi, R.; Sasaki, S.; Tsuneyuki, S. Neural-network Kohn-Sham exchange-correlation potential and its out-of-training transferability. *The Journal of Chemical Physics* **2018**, *148*, 241737.

(S8) Tozer, D. J.; Handy, N. C. The development of new exchange-correlation functionals. *The Journal of Chemical Physics* **1998**, *108*, 2545–2555.

(S9) Becke, A. D.; Johnson, E. R. A simple effective potential for exchange. *The Journal of Chemical Physics* **2006**, *124*, 221101.

(S10) Levy, M.; Zahariev, F. Ground-State Energy as a Simple Sum of Orbital Energies in Kohn-Sham Theory: A Shift in Perspective through a Shift in Potential. *Physical Review Letters* **2014**, *113*, 113002.

(S11) Verdi, C.; Karsai, F.; Liu, P.; Jinnouchi, R.; Kresse, G. Thermal transport and phase transitions of zirconia by on-the-fly machine-learned interatomic potentials. *npj Computational Materials* **2021**, *7*, 1–9.

(S12) Arthur, D.; Vassilvitskii, S. *k-means++: The Advantages of Careful Seeding*; Technical Report 2006-13, 2006.

(S13) Bystrom, K.; Kozinsky, B. CIDER: An Expressive, Nonlocal Feature Set for Machine Learning Density Functionals with Exact Constraints. *Journal of Chemical Theory and Computation* **2022**, *18*, 2180–2192.

(S14) Furthmüller, J.; Hafner, J.; Kresse, G. Dimer reconstruction and electronic surface states on clean and hydrogenated diamond (100) surfaces. *Physical Review B* **1996**, *53*, 7334–7351.

(S15) Sque, S. J.; Jones, R.; Briddon, P. R. Structure, electronics, and interaction of hydrogen and oxygen on diamond surfaces. *Physical Review B* **2006**, *73*, 085313.

(S16) Zheng, X.; Smith, P. The stable configurations for oxygen chemisorption on the diamond (100) and (111) surfaces. *Surface Science* **1992**, *262*, 219–234.

(S17) Kern, G.; Hafner, J. ***Ab initio*** calculations of the atomic and electronic structure of clean and hydrogenated diamond (110) surfaces. *Physical Review B* **1997**, *56*, 4203–4210.

(S18) Kern, G.; Hafner, J.; Kresse, G. Atomic and electronic structure of diamond (111) surfaces I. Reconstruction and hydrogen-induced de-reconstruction of the one dangling-bond surface. *Surface Science* **1996**, *366*, 445–463.

(S19) Loh, K. P.; Xie, X. N.; Yang, S. W.; Zheng, J. C. Oxygen Adsorption on (111)-Oriented Diamond: A Study with Ultraviolet Photoelectron Spectroscopy, Temperature-

Programmed Desorption, and Periodic Density Functional Theory. *The Journal of Physical Chemistry B* **2002**, *106*, 5230–5240.

(S20) Kern, G.; Hafner, J.; Kresse, G. Atomic and electronic structure of diamond (111) surfaces II. (2×1) and $(\sqrt{3} \times \sqrt{3})$ reconstructions of the clean and hydrogen-covered three dangling-bond surfaces. *Surface Science* **1996**, *366*, 464–482.

(S21) Chaudhuri, S.; Hall, S. J.; Klein, B. P.; Walker, M.; Logsdail, A. J.; Macpherson, J. V.; Maurer, R. J. Coexistence of carbonyl and ether groups on oxygen-terminated (110)-oriented diamond surfaces. *Communications Materials* **2022**, *3*, 1–9.

(S22) Furche, F. Molecular tests of the random phase approximation to the exchange-correlation energy functional. *Phys. Rev. B* **2001**, *64*, 195120.

(S23) Klimeš, J.; Kaltak, M.; Kresse, G. Predictive *GW* calculations using plane waves and pseudopotentials. *Physical Review B* **2014**, *90*, 075125.

(S24) Harl, J.; Schimka, L.; Kresse, G. Assessing the quality of the random phase approximation for lattice constants and atomization energies of solids. *Phys. Rev. B* **2010**, *81*, 115126.

(S25) Riemelmoser, S.; Kaltak, M.; Kresse, G. Plane wave basis set correction methods for RPA correlation energies. *The Journal of Chemical Physics* **2020**, *152*, 134103.

(S26) Macher, M.; Klimeš, J.; Franchini, C.; Kresse, G. The random phase approximation applied to ice. *The Journal of Chemical Physics* **2014**, *140*, 084502.

(S27) Del Ben, M.; Hutter, J.; VandeVondele, J. Probing the structural and dynamical properties of liquid water with models including non-local electron correlation. *The Journal of Chemical Physics* **2015**, *143*, 054506.

(S28) Yao, Y.; Kanai, Y. Nuclear Quantum Effect and Its Temperature Dependence in

Liquid Water from Random Phase Approximation via Artificial Neural Network. *The Journal of Physical Chemistry Letters* **2021**, *12*, 6354–6362.

(S29) Eshuis, H.; Furche, F. Basis set convergence of molecular correlation energy differences within the random phase approximation. *The Journal of Chemical Physics* **2012**, *136*, 084105.

(S30) Jinnouchi, R.; Minami, S.; Karsai, F.; Verdi, C.; Kresse, G. Proton Transport in Perfluorinated Ionomer Simulated by Machine-Learned Interatomic Potential. *J. Phys. Chem. Lett.* **2023**, *14*, 3581–3588.

(S31) Jinnouchi, R.; Lahnsteiner, J.; Karsai, F.; Kresse, G.; Bokdam, M. Phase Transitions of Hybrid Perovskites Simulated by Machine-Learning Force Fields Trained on the Fly with Bayesian Inference. *Physical Review Letters* **2019**, *122*, 225701.

(S32) Liu, P.; Verdi, C.; Karsai, F.; Kresse, G. Phase transitions of zirconia: Machine-learned force fields beyond density functional theory. *Physical Review B* **2022**, *105*, l060102.
